# Exploring the Potential of Promising Sensor Technologies for Concrete Structural Health Monitoring

**DOI:** 10.3390/ma17102410

**Published:** 2024-05-17

**Authors:** Fatheali A. Shilar, Sharanabasava V. Ganachari, Veerabhadragouda B. Patil, T. M. Yunus Khan, Abdul Saddique Shaik, Mohammed Azam Ali

**Affiliations:** 1 Department of Civil Engineering, Jain College of Engineering, Belagavi 590001, Karnataka, India; shilarone@gmail.com; 2 Center for Energy and Environment, School of Advanced Science, KLE Technological University, Hubballi 580031, Karnataka, India; 3 Institute of Energetic Materials, Faculty of Chemical Technology, University of Pardubice, 53210 Pardubice, Czech Republic; iamveerabhadraa@gmail.com; 4 Research Center for Advanced Materials Science (RCAMS), King Khalid University, P.O. Box 9004, Abha 61413, Saudi Arabia; 5 Department of Mechanical Engineering, College of Engineering, King Khalid University, Abha 61421, Saudi Arabia; ashaik@kku.edu.sa (A.S.S.); mazam@kku.edu.sa (M.A.A.)

**Keywords:** concrete sensors, steel fiber, structural health monitoring, crack, durability

## Abstract

Structural health monitoring (SHM) is crucial for maintaining concrete infrastructure. The data collected by these sensors are processed and analyzed using various analysis tools under different loadings and exposure to external conditions. Sensor-based investigation on concrete has been carried out for technologies used for designing structural health monitoring sensors. A Sensor-Infused Structural Analysis such as interfacial bond-slip model, corroded steel bar, fiber-optic sensors, carbon black and polypropylene fiber, concrete cracks, concrete carbonation, strain transfer model, and vibrational-based monitor. The compressive strength (CS) and split tensile strength (STS) values of the analyzed material fall within a range from 26 to 36 MPa and from 2 to 3 MPa, respectively. The material being studied has a range of flexural strength (FS) and density values that fall between 4.5 and 7 MPa and between 2250 and 2550 kg/m^3^. The average squared difference between the predicted and actual compressive strength values was found to be 4.405. With cement ratios of 0.3, 0.4, and 0.5, the shear strength value ranged from 4.4 to 5.6 MPa. The maximum shear strength was observed for a water–cement ratio of 0.4, with 5.5 MPa, followed by a water–cement ratio of 0.3, with 5 MPa. Optimizing the water–cement ratio achieves robust concrete (at 0.50), while a lower ratio may hinder strength (at 0.30). PZT sensors and stress-wave measurements aid in the precise structural monitoring, enhanced by steel fibers and carbon black, for improved sensitivity and mechanical properties. These findings incorporate a wide range of applications, including crack detection; strain and deformation analysis; and monitoring of temperature, moisture, and corrosion. This review pioneers sensor technology for concrete monitoring (Goal 9), urban safety (Goal 11), climate resilience (Goal 13), coastal preservation (Goal 14), and habitat protection (Goal 15) of the United Nations’ Sustainable Development Goals.

## 1. Introduction

The structural health monitoring (SHM) of concrete structures involves using sensors and other monitoring devices to continuously monitor the condition of a concrete structure and detect any signs of deterioration or damage [1]. This can include monitoring cracks, deflections, and strains in the concrete and the integrity of reinforcing steel and other structural elements. The data collected by these sensors can be analyzed to identify potential issues and help engineers decide when and how to repair or strengthen the structure. Some common techniques used in the SHM of concrete structures include ultrasonic testing, infrared thermography, and fiber-optic sensing [2].

The acoustic emissions (AEs) method involves detecting and analyzing transient stress waves generated by the sudden release of energy within a material. AE sensors are strategically placed in concrete structures to capture the high-frequency signals emitted during crack initiation, propagation, and other damage mechanisms. These signals, known as “acoustic emissions”, provide valuable insights into the location, type, and severity of structural defects, including microcracks, delamination, and corrosion-induced damage. By monitoring AE activity over time, engineers can assess the structural integrity of concrete elements and identify potential failure modes before they escalate [1,2].

Guided waves are mechanical vibrations that propagate along the surfaces or within the thickness of a material, such as concrete beams or slabs. In structural health monitoring (SHM), guided-wave techniques utilize specialized transducers to generate and detect guided wave signals, which interact with the structure and undergo amplitude, phase, and frequency changes in response to damage or defects. By analyzing the propagation characteristics of guided waves, including wave speed, attenuation, and mode conversion, engineers can identify and localize various types of structural anomalies, such as cracks, voids, and debonding. Guided wave-based SHM systems offer the advantage of long-range inspection capability, allowing for the assessment large concrete structures with minimal surface access [3,4].

The information gathered by sensors and other monitoring devices is crucial in evaluating significant structural performance parameters, like stability, structural integrity, and load-carrying capacity. Subsequently, these parameters are utilized to conclude the concrete effects of infrastructure on health conditions and performance [3]. Engineers can use this information to identify potential issues and decide when and how to restore or strengthen the structure. The sensor detects a crack in the concrete; engineers can use that information to determine the width and location of the crack and whether it poses a safety risk. Based on this information, they can decide on the appropriate action, such as making repairs or strengthening the structure [4].

To monitor concretes structural health, destructive and non-destructive methods are available. Destructive methods refer to techniques that require physically altering or damaging the structure to conduct tests: drilling holes in the concrete to extract samples for testing or breaking concrete to inspect the reinforcing steel and other structural elements. These methods can provide detailed information about the condition of the structures. Still, they are not always practical or cost-effective, and they can weaken the structure, making it more susceptible to failure [5].

Non-destructive methods, on the other hand, do not involve altering or damaging the structure. Various non-destructive methods are available for monitoring the structural health of concrete, including visual inspection, ultrasonic testing, infrared thermography, and fiber-optic sensing [5,6,7,8,9]. These methods offer information on the condition of structures without causing any alteration, making them a practical and cost-effective choice for monitoring the health of concrete structures over an extended period. They are widely used in real-time monitoring and provide data for condition assessment, trend analysis, and damage identification [7,8].

Concrete sensors can either be embedded within the structural material or bonded to the surface of the member to facilitate real-time damage assessment. Structural Surveillance Mechanisms frequently leverage discrete point sensors (such as Vibrating Wire or Fiber Bragg Grating (FBG)) or extensively spread fiber-optic sensors (DFOSs). These apparatuses are meticulously embedded within the framework of the structure. This integration method facilitates data extraction directly from the structural component instead of its superficial layers [9]. Sensors affixed to the exterior of concrete structures are typically secured through mechanical mounting rather than adhesive fixation. The potential dislodging of the sensor necessitates this precaution from the concrete surface, whether it be a discrete spot sensor or a distributed DFOS. This vulnerability is especially pronounced when the surface is subjected to external elements like rain and frost. Interestingly, the authors did not expound upon the practice of embedding sensors within grooves. This technique finds common application when installing DFOS sensors onto pre-existing concrete structures [10].

Applying acoustic sensors in cement hydration progress introduces a novel dimension to construction materials science. By harnessing the sensitivity of acoustic sensors to detect subtle acoustic emissions and vibrations within the concrete matrix, researchers and engineers gain a powerful tool to monitor and comprehend the intricate process of cement hydration. Acoustic sensors, adept at capturing even the minutest sound waves and structural oscillations, offer real-time insights into the evolving stages of cement hydration. This technology allows for the identification of critical milestones in the hydration process, such as the initial setting and final setting times, as well as the transition from the plastic to the hardened state of concrete [5,6,7,8].

The sensors are embedded directly into concrete structures to monitor moisture content, temperature, and chloride ion concentration parameters. Previous reviews have highlighted the potential of these sensors but often lacked in-depth analysis of their long-term durability, accuracy, and scalability for large-scale implementation [3,5,11,12,13].

Optical fiber sensors use light to measure strain, temperature, and other parameters in concrete structures. While previous reviews discussed the advantages of optical fiber sensing, such as high sensitivity and immunity to electromagnetic interference, they may have lacked detailed discussions on installation methods and signal-processing techniques [1,9,10,14,15,16,17].

To detect defects and damage, guided-wave techniques propagate mechanical waves through concrete structures. Past reviews have emphasized the potential of guided waves for rapid and non-destructive evaluation of concrete but may not have adequately addressed challenges related to wave dispersion and signal interpretation [18,19].

Infrared thermography utilizes thermal imaging to detect variations in surface temperature, which can indicate defects or moisture ingress in concrete. Also, highlighted the advantages of infrared thermography for the rapid inspection of large areas but may have overlooked issues such as surface emissivity variations and environmental factors affecting thermal measurements [20,21,22].

A Vibration analysis involves monitoring the dynamic response of concrete structures to detect changes indicative of damage or deterioration. While prior reviews have discussed the principles of vibration analysis and its applications in SHM, they may have lacked detailed discussions on sensor placement optimization and data interpretation techniques [23,24,25,26,27,28]. Acoustic emission sensors detect transient stress waves generated by internal damage mechanisms within concrete structures. Also, highlighted the sensitivity of acoustic emission techniques to early-stage damage but may not have fully addressed challenges such as background noise interference and source localization accuracy [29,30,31,32].

Moreover, the non-invasive nature of acoustic sensing ensures that the structural integrity of the cement matrix remains uncompromised during analysis. This makes it a valuable asset for monitoring in situ conditions and even providing early warnings regarding potential anomalies or irregularities in the hydration process. Constant and on-site monitoring of the surface attributes of concrete in specific exposure conditions can be a helpful means of accurately estimating deterioration rates and the service performance of structures [14]. This is because the concrete surface is susceptible to various environmental factors, including temperature, humidity, and exposure to chemicals, which can cause changes in the surface properties and ultimately lead to concrete deterioration [15].

**Figure 1:**
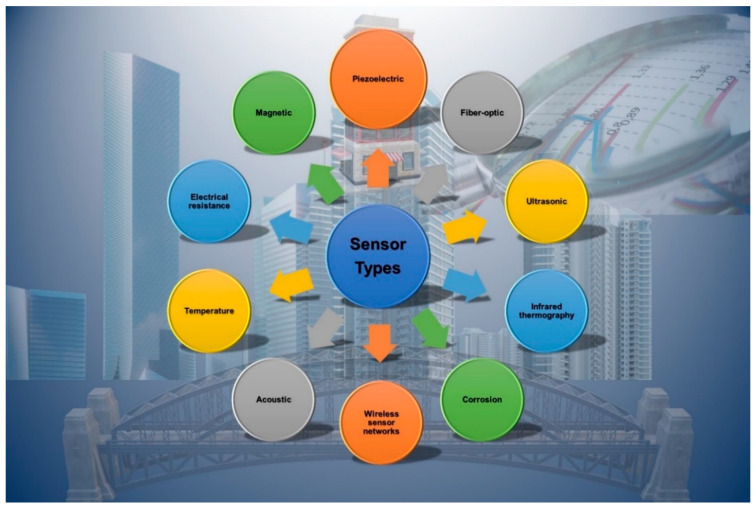
Schematic of measurement techniques employed in sensors.

Ongoing monitoring of the concrete surface characteristics can yield crucial data regarding the structures performance and deterioration rate over time. Monitoring the surface pH, chloride content, and temperature can provide important information about the degree of carbonation, chloride penetration, and freeze–thaw cycles, respectively, which are critical for the durability of the concrete [18]. By monitoring these parameters continuously, engineers can detect any changes in the surface properties early on and take appropriate measures to prevent further deterioration. In situ monitoring of the surface characteristics of concrete allows for a more realistic assessment of the condition of structures because it allows researchers to monitor the structure in its actual environment rather than in a laboratory. This provides more accurate data and gives engineers a better understanding of how the structure is performing in the field [20]. Figure 1 shows a graphical representation of sensor technology in construction. This study presents a relatively straightforward and concise covalent immobilization method for developing a sensor capable of monitoring.

The ongoing and on-site monitoring of concrete surface characteristics under particular exposure conditions can furnish valuable insights into the condition of the structure, enabling engineers to make more precise predictions regarding its durability and service performance. Sensors can gather a broad spectrum of concrete data throughout their lifecycle, including curing progress, daily performance, appearance, and corrosion progression [23]. During the curing process of the concrete mixture, the strains related to the shrinkage of the concrete and the cracks formed in the concrete are also measured. This provides important information about the stability of the structure and load-carrying capacity and is used to detect any signs of damage or deterioration [24]. Sensors are used to monitor the appearance of the concrete and the progress of corrosion. Sensors were used to measure the pH of the concrete surface and detect the presence of chlorides, indicating the beginning of corrosion of the reinforcing steel [25,29].

This current review article aligns with several of the United Nations Sustainable Development Goals (SDGs):

Goal 9: Industry, Innovation, and Infrastructure—The current article explores innovative sensor technologies for monitoring the health of concrete structures, contributing to the advancement of infrastructure monitoring and maintenance practices.

Goal 11: Sustainable Cities and Communities—By enhancing the monitoring and maintenance of concrete structures, your review supports the creation of safer, more resilient cities and communities.

Goal 13: Climate Action—Effective structural health monitoring can help identify vulnerabilities and mitigate risks associated with climate change impacts on concrete infrastructure, contributing to climate resilience.

Goal 14: Life Below Water—Monitoring the structural health of concrete infrastructure helps prevent the deterioration of coastal structures, reducing the risk of marine ecosystem damage and preserving underwater habitats.

Goal 15: Life on Land—By maintaining the integrity of concrete structures, your review indirectly supports land conservation efforts, as well-designed infrastructure minimizes the need for land-use changes and habitat destruction.

Around 200+ article sensors were studied with keywords such as “infrared thermography”, “optical fiber sensing electromagnetic methods”, “corrosion sensor”, “infrared thermography”, “optical fiber sensing”, “electromagnetic methods”, “corrosion sensors”, “thermocouples”, etc. Data about the sensor used in concrete structures throughout its lifecycle were analyzed using a timeline series decomposition, component analysis, seasonal analysis, and density plot.

## 2. Technologies Used for Designing Structural Health Monitoring Sensors

The structural health monitoring (SHM) of concrete involves various basic methods to assess condition of concrete structures, integrity, and performance. Visual assessment is one of the primary methods used in SHM, involving inspecting concrete surfaces for cracks, spalling, delamination, and other visible signs of distress. Non-destructive testing techniques allow for the evaluation of concrete without causing damage. Ultrasonic testing uses high-frequency sound waves to detect internal flaws and voids and measure concrete thickness. Ground-penetrating radar utilizes electromagnetic radiation to map subsurface features, including rebar corrosion, voids, and delamination. Impact-echo testing is used to analyze the frequency response of stress waves that impact the concrete surface to detect flaws and estimate thickness [33,34].

Embedded sensors, such as strain gauges, accelerometers, and piezoelectric transducers (PZTs), are installed within concrete structures to monitor parameters like strain, vibration, and acoustic emissions, providing real-time data on structural behavior. Acoustic emission monitoring detects the release of transient stress waves caused by internal damage or crack propagation within the concrete, offering insights into structural integrity and damage progression. Electrical resistance-based techniques assess changes in electrical properties of concrete due to moisture ingress, chloride penetration, and rebar corrosion, providing indications of concrete deterioration. Infrared thermography detects temperature variations on concrete surfaces, identifying areas of potential moisture ingress, delamination, or voids. Corrosion-monitoring techniques such as electrochemical impedance spectroscopy (EIS) and half-cell potential measurement are employed to monitor the corrosion activity of embedded reinforcement within concrete structures, helping to prevent corrosion-induced deterioration [29,34].

**Figure 2:**
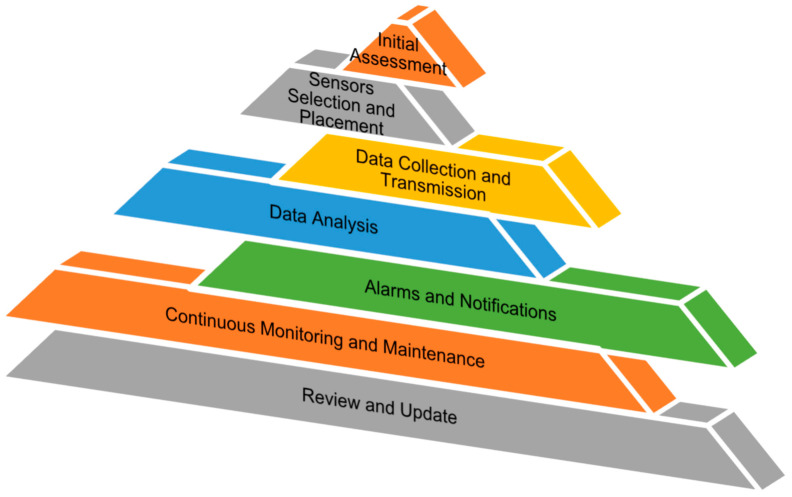
Chart representing investigation of structural health monitoring.

Several technologies are commonly used for designing structural health monitoring sensors for concrete structures. Figure 2 depicts a flowchart of the investigation of structural health monitoring. The first step of the process is the initial assessment of the structure to be monitored, which includes its design, materials, loading conditions, and operating environment. Then, the appropriate sensors and placement locations are chosen based on the type of damage and failure mechanisms expected, as well as the desired level of accuracy and reliability of the monitoring system [30]. The sensors are installed and activated, and the data collection and -transmission system is configured to send data to a central monitoring location. The data received from the sensors are then processed and analyzed to extract relevant information about the structure state, including the presence and location of any damage or anomalies [31]. After that, a threshold for the damage and failure indicators is established, and an alarm and notification system is set up to alert the relevant stakeholders if the thresholds are exceeded. The structure is continuously monitored, and maintenance and repairs are performed as needed, based on the data analysis and alarms results. The SHM system is regularly reviewed and updated, including the sensors, data-collection and -analysis methods, alarms and notifications, and maintenance procedures, to ensure its effectiveness and adapt to changes in the structure or operating environment [33,34]. Table 1 shows the comparison of various sensors and techniques used in concrete.

### 2.1. Fiber-Optic Sensor

Fiber-optic sensors employ optical fibers to detect variations in several physical parameters, including temperature, pressure, strain, and displacement [42]. They work by measuring the amount of light transmitted through the optical fiber, which is affected by the measured physical parameter [43]. Fiber-optic sensors are highly sensitive and can detect small changes in the physical parameters. They are also immune to electromagnetic interference, making them suitable for use in harsh environments [44,45]. This change in light transmission can then be detected and used to infer the value of the physical parameter. Fiber-optic sensors offer significant advantages, such as high sensitivity, immunity to electromagnetic interference, and the capacity to function in challenging environments. They are used in various fields, such as telecommunications, medicine, aerospace, oil and gas, structural monitoring, and many more [47,48].

### 2.2. Ultrasonic Sensor

**Figure 3:**
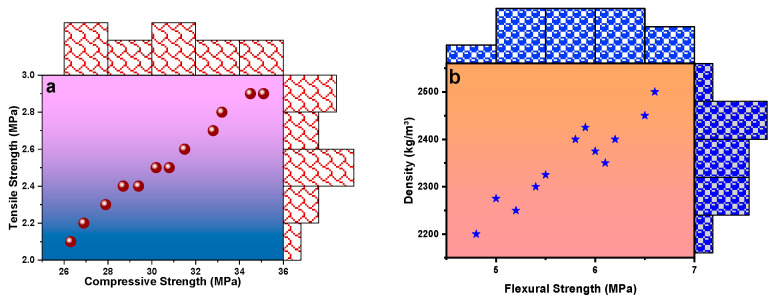
Co-relation between (**a**) CS vs. STS and (**b**) FS vs. density.

**Figure 4:**
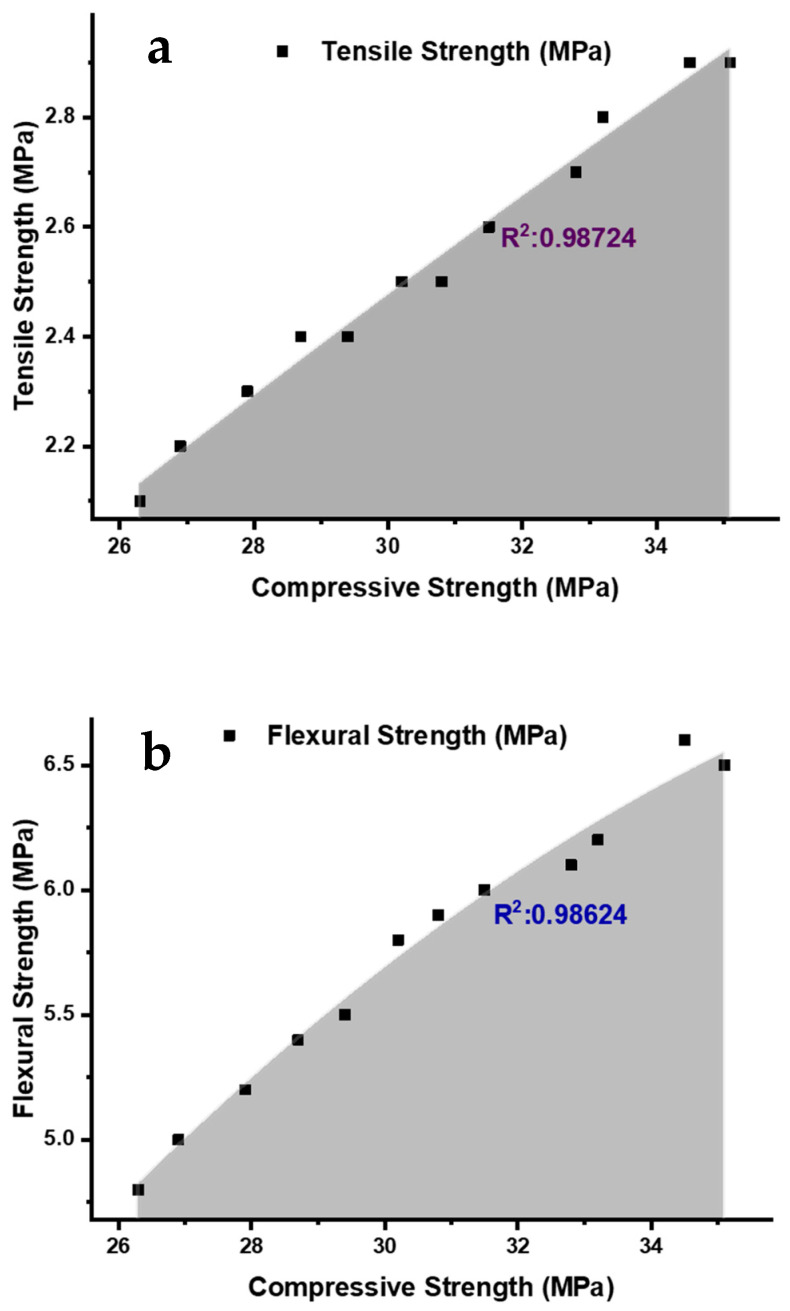
Regression analysis between (**a**) CS vs. STS and (**b**) CS vs. FS.

Ultrasonic sensors emit an ultrasonic wave and measure the time it takes for the wave to reflect (the time of flight) after hitting an object [49]. Ultrasonic sensors have a range of applications, including distance measurement, level sensing, and object detection and avoidance in robotics [50]. Ultrasonic sensors have good accuracy and a long range and can work in various environments and conditions, including harsh and extreme environments [51]. Ultrasonic sensors monitor bridge health by measuring the distance between the sensor and the surface [52]. Ultrasonic sensors inspect tunnel conditions by detecting cracks, voids, and other defects in the tunnel walls. The advantages of ultrasonic sensors in civil engineering are that they are non-destructive, easy to use, and provide real-time data [53,54,55]. Utilizing data from prior literature [10,14,15,18,56,57,58,59,60,61,62,63,64,65] wherein researchers employed ultrasonic sensors to examine strength parameters, an exploration was conducted into the correlation between compressive strength (CS) and shear strength (ST), as well as flexural strength (FS) and density. Figure 3 shows the correlation between (a) CS and STS, and (b) FS and density. The compressive strength (CS) and split tensile strength (STS) values of the analyzed material fall within a range from 26 to 36 MPa and from 2 to 3 MPa, respectively. The material being studied has a range of flexural strength (FS) and density values that fall between 4.5 and 7 MPa and between 2250 and 2550 kg/m^3^, respectively (refer to Figure 3b). It has been found that there is a linear relationship between the FS and density values. These sensors use sound waves to detect changes in the concrete, such as cracks, delamination, and corrosion. A regression analysis between (a) CS and STS and (b) CS and FS is presented in Figure 4. The regression analysis conducted for the CS and STS of materials; values yielded an R^2^ value of 0.98724. This indicates that 98.72% of the variation in CS can be explained by changes in STS, and vice versa. The high R^2^ value signifies a strong linear relationship between the two variables, suggesting that changes in one variable are strongly associated with changes in the other (refer to Figure 3a). The regression analysis performed for the CS and FS values of the material being studied resulted in an R^2^ value of 0.98624. This indicates that 98.62% of the variation in CS can be explained by changes in FS, and vice versa. The high R2 value signifies a strong linear relationship between the two variables. The data were obtained from previous literature, i.e., References [5,14,18,39,47,66,67,68,69,70,71,72,73,74], to plot Figure 3 and Figure 4. 

### 2.3. Corrosion Sensor

Corrosion in reinforcing steel is detected by measuring the electrical resistance between the steel and the concrete. This method, known as half-cell potential testing, measures the voltage difference between the steel and a reference electrode, such as a copper/sulfate electrode, embedded in the concrete [75]. These sensors can detect corrosion in the reinforcing steel by measuring the electrical resistance between the steel and the concrete or by measuring the potential difference between the steel and a reference electrode [66]. A decrease in electrical resistance and an increase in potential difference indicate corrosion activity in the steel, because, as corrosion occurs, the steel corrodes and becomes more conductive, leading to decreased resistance. Additionally, as corrosion occurs, the steel becomes more negative about the reference electrode, increasing potential difference. One additional method for detecting the presence of corrosion in reinforcing steel is to measure the potential difference between the steel and a reference electrode. This method is known as impressed current cathodic protection [67]. Several types of corrosion sensors work on different principles; for instance, the linear polarization resistance (LPR) sensor works by passing a small direct current through the steel and measuring the resistance of the steel. The resistance of the steel changes as the steel corrodes. Electrochemical noise (EN) sensors measure the random voltage fluctuations that occur on the steel surface due to corrosion. Half-cell potential (HCP) sensors measure the potential difference between the steel and a reference electrode embedded in the concrete [68]. As corrosion occurs, the steel becomes more negative about the reference electrode, increasing the potential difference. Impedance spectroscopy (IS) sensors measure the complex impedance of the steel as a function of frequency. As corrosion occurs, the impedance of the steel changes. The composition of the steel and the surrounding environment also affect the working principle of the corrosion sensors. The working principle of a corrosion sensor is to detect corrosion by measuring the changes in the steel electrical, chemical, or physical properties [69,70,71,76].

### 2.4. Cement-Based Sensors 

Piezoresistivity is a phenomenon that achieves self-sensing in cement-based sensors by distinguishing between reversible and irreversible behaviors. The sensitivity of piezoresistivity can be measured using the gauge factor [1,8]. AC impedance spectroscopy (ACIS) is a promising non-destructive technique that can study the microstructural composition of cement-based sensors. Many researchers have been recently used it and can provide valuable insights into the microstructural characterization of these sensors [47,48,49,50,51]. The fundamental concepts of piezoresistivity and ACIS include the piezoresistivity theory, resistance measurement methodology, strain/damage sensing, theories of conduction, the AC impedance spectroscopy theory, and equivalent circuit models [57,58,59]. The direct-current four-pole method based on embedded gauze electrodes is suitable for measuring the resistance of piezoresistive cement-based sensors (PCSs). Combining carbon fiber and carbon black can improve the piezoresistivity of cement-based materials, enhancing PCS’s sensitivity, linearity, and repeatability. The piezoresistive responses of the sensors are almost unaffected by the loading rate when it is lower than 0.20 cm/min, but the effect increases with higher loading rates [72,73,74,77,78,79,80]. CNT-filled cement-based sensors exhibit stable and reversible piezoresistive responses within the elastic regime, with a sensitivity of 0.911 Kω/Mpa, linearity of 7.16%, repeatability of 1.53%, and hysteresis of 7.24%. This paper’s novelty lies in using CNTs as conductive fillers in fabricating piezoresistive cement-based sensors, with a CNT content level of about 0.1, which is lower than other conductive fillers used in previous studies [80,81].

The electro-mechanical impedance (EMI) or admittance method has applications in the concrete curing and the early-age behavior of cementitious materials. The process involves the utilization of piezoelectric transducers (PZTs) to measure the mechanical impedance or admittance of concrete specimens. This measurement provides valuable insights into the internal microstructure and mechanical properties of material during the curing process and the early stages of hydration. The mechanism underlying the EMI method lies in the interaction between the PZT transducers and the concrete specimen. When a voltage is applied to the PZT, it propagates stress waves through the material. These waves encounter interfaces between different phases of the concrete, such as aggregates, cement paste, and voids. The impedance or admittance measured by the transducer reflects the interaction of these stress waves with the concrete microstructure, offering information about the stiffness, density, and hydration state of the material. Past results from research on EMI applications in concrete curing and early-age monitoring have demonstrated its effectiveness in several key areas. Firstly, the method enables real-time monitoring of the curing process, allowing for adjustments to be made to optimize concrete quality and strength development. Additionally, EMI has been utilized to assess the homogeneity of concrete mixtures and detect any anomalies or defects that may arise during curing. Furthermore, studies have shown that EMI measurements correlate well with traditional methods of assessing concrete properties, validating EMI use as a reliable monitoring technique. The EMI or admittance method offers a non-destructive and sensitive approach to evaluating curing process of cementitious materials and early-age behavior [47,51].

The statical values, minimum, and maximum values of the impedance are key parameters in assessing the condition of the concrete specimen. The impedance values reflect the mechanical properties and integrity of the material, with deviations from expected values indicating potential defects or changes in the concrete structure. Impedance is typically measured in units of ohms (Ω) and is quantified as the ratio of the applied voltage to the resulting current in the PZT transducer. By monitoring changes in impedance over time, researchers can analyze the evolution of mechanical properties, such as stiffness and damping, and detect the presence of defects, like cracks or voids, within the concrete specimen. The EMI method allows for the visualization of impedance data in various formats, such as impedance spectra or impedance maps, providing detailed insights into the spatial distribution and magnitude of changes within the concrete structure. This comprehensive analysis enables researchers to identify areas of concern and tailor maintenance or repair strategies accordingly, ultimately enhancing the durability and performance of concrete infrastructure [49,52].

### 2.5. Thermocouples

A thermocouple is a type of temperature sensor that operates on the principle of the Seebeck effect. It consists of two dissimilar metal wires joined at both ends to form two junctions, a hot junction and a reference junction. When the temperature of the hot junction changes, a small voltage is generated between the two junctions, which is proportional to the temperature difference between them. This voltage can be measured and used to infer the temperature of the hot junction [82]. The reference junction is typically kept at a constant temperature, serving as a temperature measurement baseline. Thermocouples are commonly used in various industrial, scientific, and engineering applications to measure temperatures ranging from very low to very high [75,83]. The voltage generated by the thermocouple can be measured using a thermocouple meter or an instrument called a thermocouple amplifier, which amplifies the small thermocouple voltage to a level that can be read by a standard voltmeter [84]. Thermocouples are widely used in research, industrial, and domestic applications because of their low cost, high accuracy, and wide range of temperature measurements. They are available in many different types, each made of metal combinations and suitable for specific temperature ranges and environments [85].

### 2.6. Resistance Temperature Detectors (RTDs)

**Figure 5:**
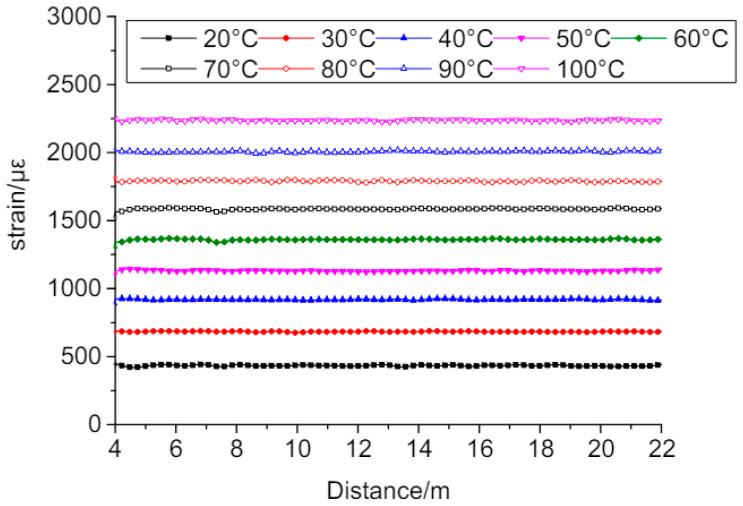
Temperature calibration test results of the tight sheath strain sensing fiber (reproduced from) [91].

Resistance temperature detectors (RTDs) are made up of a wire composed of a material with a known and predictable change in resistance as temperature varies, such as platinum, copper, or nickel. RTD are sensors used to measure temperature by measuring the electrical resistance of a material [86]. RTDs have a high measurement accuracy, typically within a 0.1 to 0.5 °C range. RTDs measure temperatures from −200 to 850 °C, making them suitable for various applications. RTDs are relatively stable and repeatable over time, which makes them ideal for long-term monitoring of concrete structures [87]. RTDs can be used to measure temperature non-destructively, avoiding the need for costly and disruptive repairs. The temperature calibration test results of the tight-sheath strain-sensing fiber are presented in Figure 5. The results are significant, as they provide information on the accuracy and reliability of the fiber in measuring strain under different temperature conditions. The test results can be used to calibrate the fiber for use in practical applications where temperature changes may occur. The calibration process involves adjusting the fiber output to account for temperature changes, thereby improving strain measurement accuracy [88]. RTDs can be easily installed in concrete structures, and the temperature measurements can be taken remotely using a data logger or a wireless sensor network. RTDs can be used for long-term monitoring of concrete structures, providing valuable data for assessing the remaining service life of the structure [89]. RTDs are significant in concrete because of their non-destructive nature, high accuracy, wide temperature range, stability, ease of installation and long-term monitoring abilities that can help assess the structures remaining service life and predict potential failures [90].

### 2.7. Thermistor

Thermistors are temperature-sensitive resistors that can measure temperature in concrete structures. They are made of semiconductor materials with a significant change in resistance with a small temperature change. The resistance of a thermistor decreases as the temperature increases, allowing the change in resistance to be used to infer the temperature [92]. Thermistors are relatively low-cost compared to other temperature sensors, making them a cost-effective option for monitoring concrete structures. Small thermistors can be easily integrated into concrete structures, particularly useful for monitoring hard-to-reach areas [93]. Thermistors are used to measure temperature non-destructively, avoiding the need for costly and disruptive repairs.

Thermistors can be easily installed in concrete structures, and the temperature measurements can be taken remotely using a data logger or a wireless sensor network. Thermistors are used for long-term monitoring of concrete structures, providing valuable data for assessing the remaining service life of structures. Data collected by thermistors are used to predict potential failures in concrete structures and enable maintenance before the actual failure [94]. Thermistors, due to their high sensitivity, low cost, small size, and non-destructive nature, are often used in concrete structures as a sensor. Its long-term monitoring abilities and ability to predict potential failures make it a valuable asset in assessing the remaining service life of the structure [95].

### 2.8. Near-Infrared Spectroscopy

This method uses the principle of absorption of specific wavelengths of light by cement hydration products. The change in the absorption can be used to infer the degree of hydration. Near-infrared (NIR) spectroscopy works on the principle of absorption of light in the NIR range (typically 750–2500 nm) by a sample [96]. When light at a specific wavelength is directed at a sample, the sample absorbs some of the light while the rest is transmitted or reflected. By measuring the intensity of the light transmitted or reflected at different wavelengths, it is possible to determine the composition and concentration of the chemical compounds in the sample. The absorption of light by a sample is specific to the compound’s chemical structure, allowing for the identification and quantification of the compounds present. The non-destructive technique can be employed for various applications, including food and agricultural analysis, medical diagnostics, and industrial process control [97,98,99].

NIR spectroscopy has numerous applications in civil engineering, such as analyzing the composition and quality of cement and concrete. This technique measures water content, cement content, and aggregate type. NIR spectroscopy is a non-destructive method that can be applied to various fields, including food and agricultural analysis, medical diagnostics, and industrial process control. NIR spectroscopy analyze soil chemical and physical properties, including the measurement of organic matter, clay content, and pH. NIR spectroscopy can analyze the composition and quality of asphalt, including the measurement of binder content, aggregate type, and contaminants [100,101]. NIR spectroscopy is used for non-destructive testing of structures, including of measuring moisture content in wood and the detection of cracks and delamination in concrete and other building materials. NIR spectroscopy is used in quality control processes to ensure construction materials meet the required standards and specifications. NIR spectroscopy used to monitor environmental pollutants, such as heavy metals and organic compounds in soil, water, and air. NIR spectroscopy is a powerful tool that can provide fast, accurate, and non-destructive analysis of a wide range of civil engineering materials and structures, allowing for better quality control, improved materials selection, and the optimization of construction processes [96,102,103,104].

### 2.9. Infrared Thermography

Infrared thermography sensors use infrared radiation to measure the temperature of an object. The sensor detects the infrared radiation emitted by the object and uses this information to infer the temperature of the object [105]. It is a non-contact method of temperature measurement, which means it does not require physical contact with the object being measured. Instead, it uses a camera to detect and measure the infrared radiation emitted by the object [60]. Infrared thermography allows for non-contact temperature measurement, which is particularly useful for monitoring concrete structures that are difficult to access or would be damaged by contact sensors. Infrared thermography is used to measure temperature non-destructively, avoiding the need for costly and disruptive repairs [70]. Infrared thermography is easily installed in concrete structures, and the temperature measurements are taken remotely, using a camera or a wireless sensor network. Infrared thermography is used for long-term monitoring of concrete structures, providing valuable data for the assessment of the remaining service life of the structure [97]. Data collected by infrared thermography can be used to predict potential failures in concrete structures and enable maintenance before the actual failure [96].

The utilization of infrared thermography sensors unveils a multidimensional analysis encompassing three key methodologies: timeline series decomposition, component analysis, and seasonal analysis. In a timeline series decomposition, by harnessing the power of timeline series decomposition, the compressive strength results are meticulously dissected into constituent elements. This process allows for the disentanglement of intricate trends and patterns that might otherwise remain obscured. Through this method, the interplay of various factors contributing to compressive strength can be discerned with a newfound clarity. A component analysis adds an extra layer of granularity to exploring compressive strength outcomes. It delves into the distinct constituents shaping the overall results, unraveling the specific contributions of individual components. This approach proves invaluable in pinpointing the strengths and weaknesses within the structural matrix, ultimately guiding the formulation of precision-driven strategies.

**Figure 6:**
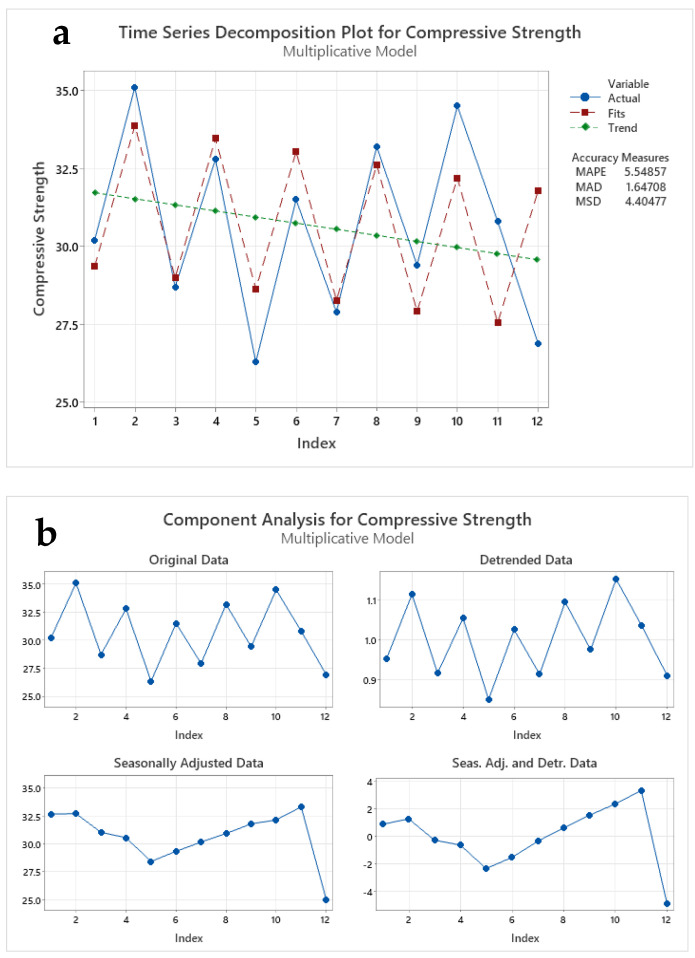

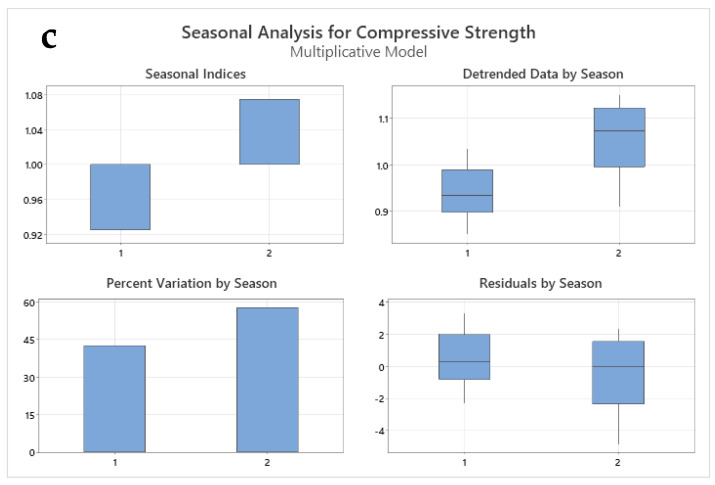
CS results analysis using (**a**) timeline series decomposition, (**b**) component analysis, and (**c**) seasonal analysis.

Seasonal analysis: As the seasons unfold, so does the impact on compressive strength. facilitated by infrared thermography sensors, sheds light on the influence of changing environmental conditions. This meticulous scrutiny of how compressive strength responds to varying seasons engenders pivotal insights for both short-term adjustments and long-term structural fortification. In harnessing the capabilities of infrared thermography sensors, the domain of compressive strength analysis takes on a new dimension. With their ability to capture and translate thermal signatures into meaningful data, these sensors empower structural analysts to navigate the intricate landscape of compressive strength with unprecedented precision and insight. The analysis of CS results using (a) time series decomposition, (b) component analysis, and (c) seasonal analysis is presented in Figure 6. For the Figure 6 plot, the data were obtained from previous literature, i.e., [5,14,18,39,47,66,67,68,69,70,71,72,73,74]. A time-series decomposition involves breaking down the CS results into trend, seasonal, and residual components. This technique can provide insights into the long-term behavior of the CS values and can help identify any recurring patterns or trends in the data. The component analysis identifies the factors contributing to the CS results [103]. This technique helps determine which variables have the most significant impact on the CS values, allowing for more targeted interventions to improve the strength of the material (refer to Figure 6b). The seasonal analysis involves examining the CS results for any seasonality or cyclical patterns that may occur over specific time periods. This technique is useful in predicting when the CS values will likely increase or decrease based on past patterns refer Figure 6c. Applying these three techniques to analyze the CS results provides a comprehensive understanding of the data. It helps to identify any underlying trends, patterns, or factors affecting the strength of the material [104]. Infrared thermography is a non-contact, non-destructive, and easy-to-install temperature measurement method that can be used to measure the temperature of concrete structures with a high accuracy and wide temperature range. It is particularly useful for monitoring concrete structures that are difficult to access and used for long-term monitoring and predictive maintenance [60].

An analysis of compressive strength values for three different variables is offered in Figure 6a. The variables mentioned are “actual”, “fits”, and “trending line”, and the analysis incorporates certain key performance indicators such as MAPE (Mean Absolute Percentage Error), MAD (Mean Absolute Deviation), and MSD (Mean Squared Deviation). MAPE is a measure of the prediction accuracy of a forecasting method, often used in the context of time series analysis. It calculates the average percentage difference between the forecasted and the actual values. In this case, the MAPE value of 5.54857 indicates the average percentage error between the predicted and actual values for the compressive strength. MAD is a statistical measure that calculates the average of the absolute deviations of a set of values from their mean. It gives an insight into the variability within the dataset. The value of 1.64708 represents the average absolute difference between the individual compressive strength values and their mean. MSD is a statistical measure that calculates the average of the squares of the differences between the predicted and actual values. It helps us understand the average magnitude of the errors or deviations. The value of 4.40477 is the average squared difference between the predicted and actual compressive strength values.

In this context, the “fits” likely refer to the fitted values obtained from a model, while the “trending line” might indicate a line representing the trend or pattern in the compressive-strength data. Further interpretation and analysis of these values would depend on the specific context and purpose of the analysis and the field or industry to which these variables and measurements relate.

The technology uses infrared cameras to detect concrete temperature changes, indicating structural distress, such as cracking or corrosion [100]. These sensors detect and convert the infrared radiation emitted by an object into a visual image that displays the temperature distribution across the surface. In civil engineering, IT sensors are used for various applications; they detect heat leaks in buildings and help identify areas where energy is wasted [106]. The IT sensor detect areas of a structure experiencing excessive heat, which can indicate structural damage or deterioration. IT sensors detect hot spots in electrical and mechanical equipment, indicating potential problems before they lead to equipment failure. IT sensors are used to detect hot spots in buildings, which can indicate the presence of a fire [107]. IT sensors are used to detect the temperature distribution of concrete to identify any defects or cracks in the concrete [101,102,108]. The advantage of using IT sensors in civil engineering is that they can provide real-time data that can be used in difficult-to-access locations and in harsh environments. They also can detect hidden defects, which can help identify potential problems before they lead to equipment failure or structural damage [109,110].

### 2.10. Acoustic Sensor

Acoustic sensors measure the ultrasonic wave velocity through the cement as it hydrates. The change in wave velocity can be used to infer the degree of hydration. Acoustic sensors, also known as ultrasonic sensors, are used in civil engineering to measure and monitor various physical parameters, such as distance, displacement, and velocity [111].

### 2.11. Specific Applications of Acoustic Sensors in Construction

Acoustic sensors monitor the structural integrity of bridges, buildings, and other structures. The sensors can detect changes in the sound waves emitted by the structure, indicating changes in the structure, such as cracking or deformation [112].

Concrete testing: Acoustic sensors can be used to test the quality of concrete. The sensors can detect the velocity of sound waves passing through the concrete, which can be used to infer the strength and density of the concrete.

Leak detection: Acoustic sensors can detect leaks in pipelines and underground tanks. The sensors can detect the sound of the leak, which can be used to locate the source of the leak [113]. Acoustic sensors are used to test the properties of soil. The sensors can detect the velocity of sound waves passing through the soil, which can be used to infer the density and stiffness of the soil.

Non-destructive testing: Acoustic sensors can test the quality of various materials, such as metals and composites, without causing any damage [114]. 

Slope stability monitoring: Acoustic sensors can be used to monitor slope stability. The sensors can detect changes in the sound waves emitted by the slope, which can indicate changes in the slope such as cracking or deformation. Acoustic sensors are non-invasive and can provide real-time data, which can be used to adjust the structure or material as needed. They can also provide long-term data, which can be used to track changes over time and predict future problems [115].

The working principle of acoustic sensors, also known as ultrasonic sensors, is based on measuring sound waves. These sensors emit and detect high frequency sound waves, typically above the range of human hearing (20 kHz) [116]. The working principle of an acoustic sensor can be broken down into three steps, the first step is emission: Acoustic sensors emit a sound wave, typically in the form of a short pulse, into the environment. This sound wave can be in the form of a continuous wave or a pulsed wave. The sound wave is typically in the ultrasonic frequency range, above the range of human hearing. The second step involves the propagation of a sound wave through the environment and its interactions with the objects or materials that it encounters. The sound wave is reflected by the objects or materials, and the reflection is captured by the sensor. The third step is detection: The sensor detects the reflected sound wave and measures various parameters, such as the amplitude and the time of flight of the sound wave. These measurements can infer various physical parameters such as distance, displacement, and velocity [98,113,114].

The working principle of an acoustic sensor can be summarized as the sensor emits a sound wave (ultrasonic). The sound wave propagates and reflects from the object or material. The sensor detects the reflected wave and measures the parameters. The data are processed to infer the distance, displacement, and velocity [117]. It is worth noting that the sensor’s range, accuracy, and resolution depend on the frequency and power of the emitted sound wave, the properties of the material and the environment, and the type of sensor and its design [118].

### 2.12. Electrical Resistance Sensor

**Figure 7:**
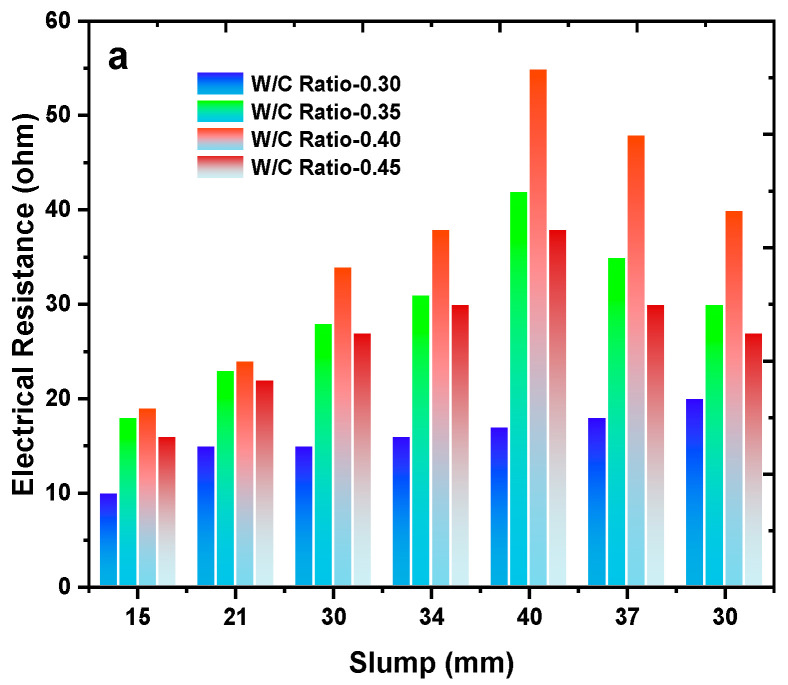

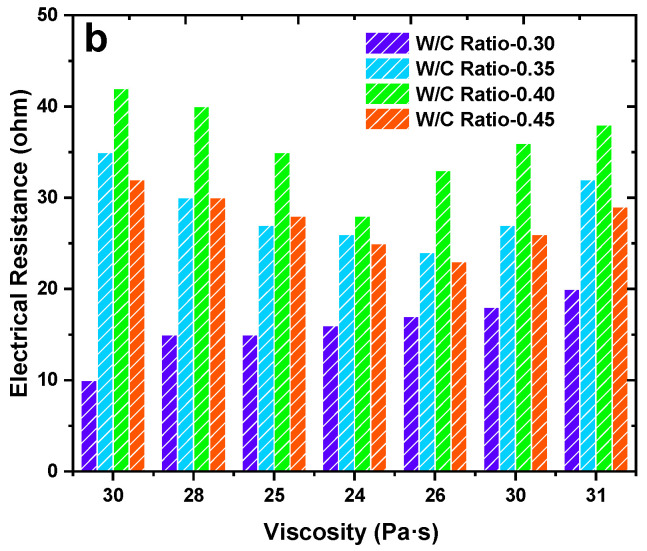
Electrical resistance analysis: (**a**) slump and (**b**) viscosity.

The electrical resistance analysis for (a) slump and (b) viscosity. The electrical resistance of the material under study ranges from 0 to 60 ohms. In contrast, the slump values range from 15 to 60 mm, depending on the water-to-cement (W/C) ratio, which varies from 0.30 to 0.45, shown in Figure 7. For a W/C ratio of 0.40, the highest slump value of 40 mm is observed when the electrical resistance is 55 ohms. As the W/C ratio increases, the slump value and electrical resistance increase up to a W/C ratio of 0.40. However, beyond this point, a decline in performance (viz., Figure 7a).

**Figure 8:**
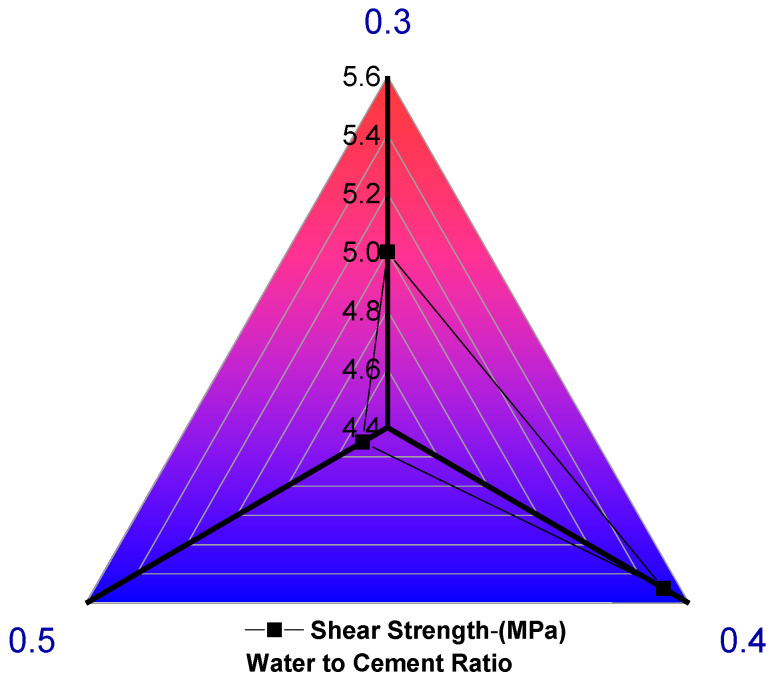
Co-relation between various ratios of W/C and shear strength.

These findings have significant implications for the manufacturing and application of the material. By controlling the W/C ratio and electrical resistance, materials with desired slump values are critical for specific applications [119]. Additionally, understanding the relationship between W/C ratio and electrical resistance can help optimize the performance of material, ensuring that it meets the specifications for different applications. The electrical resistance of the material under study ranges from 0 to 50 ohms. In contrast, the viscosity values range from 24 to 31 Pa. s, depending on the water-to-cement (W/C) ratio, which varies from 0.30 to 0.45. For a W/C ratio of 0.40, the highest viscosity value of 31 Pa. s is observed when the electrical resistance is 43 ohms (refer to Figure 7b). This finding is significant as it highlights the importance of controlling the W/C ratio and electrical resistance to achieve desired viscosity values. Understanding the relationship between W/C ratio, electrical resistance, and viscosity is critical in optimizing material performance for various applications. The data investigation was performed using sensor, and the gathered results were visualized through plots by referring [37,48,51,60,61,62,63,64,76,120,121,122,123,124,125,126,127,128,129,130]. For different water-cement ratios, the shear strength value variation is analyzed in Figure 8. W/C of 0.3, 0.4 and 0.5 ratios, the shear strength value ranged from 4.4 to 5.6 MPa. Maximum shear strength was observed for a W/C ratio of 0.4, with 5.5 MPa, followed by a W/C ratio of 0.3, with 5MPa. The least strength was observed for a W/C ratio of 0.5.

Electrical resistivity sensors, for instance, provide information about the internal moisture content of concrete, aiding in durability-assessment and corrosion-prevention strategies. By measuring the electrical conductivity variations, these sensors provide insights into the levels of moisture present within the material [131]. This sensor assesses the internal moisture content and conductivity, which are directly linked to the susceptibility to cracking, freeze thaw damage, and other forms of deterioration over time of the material. Sudden changes in electrical conductivity can signal potential issues, like the ingress of harmful chemicals or the onset of structural problems, enabling timely interventions [132,133]. Also, by monitoring conductivity changes, they provide valuable insights into the presence of corrosive agents that might compromise the structural integrity. The sensors also facilitate the monitoring of health of concrete structures [134]. By tracking changes in electrical properties, such as resistance, the sensors can detect and quantify structural damage or deterioration, allowing for informed decisions on maintenance and repairs [135,136]. Utilizing electrical resistance sensors contributes to optimizing concrete mix designs and construction practices [137,138]. Real-time data on moisture levels and conductivity guide adjustments in the curing process, enhancing the overall performance and longevity of the concrete structure.

### 2.13. Calorimetry

Calorimetry can be used to measure the heat generated during the hydration process, which can be used to infer the degree of hydration [84]. The amount of heat produced during the hydration process depends on the degree of hydration, representing the extent of the reaction between cement and water [73,82,84]. The water–cement (W/C) ratio is a crucial parameter in concrete mix design that determines the concrete’s strength, durability, and workability. A W/C ratio of 0.30 to 0.50 indicates the range of water–cement ratios used for creating concrete of different strengths and consistencies.

**Figure 9:**
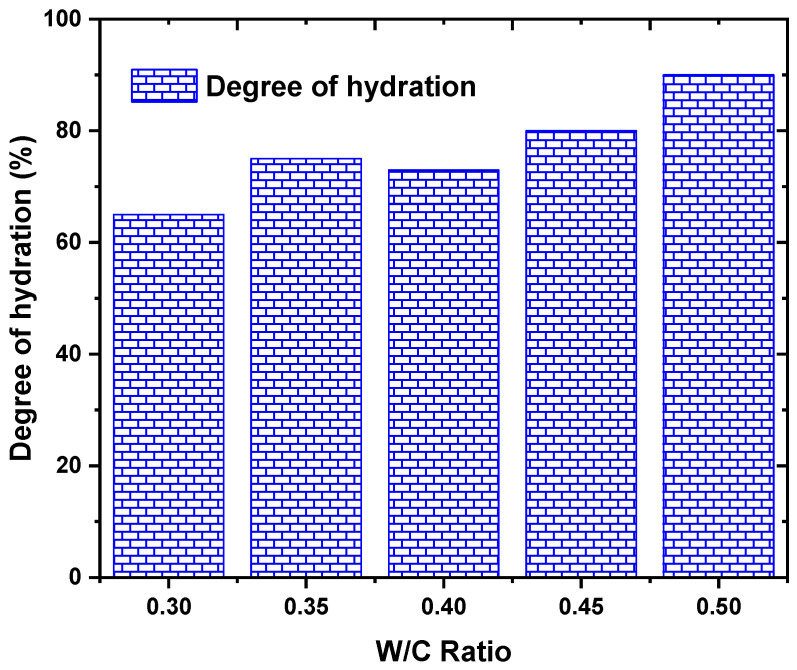
Effect of different W/C ratios on the degree of hydration.

The degree of hydration refers to the extent to which the cement particles react with water in the concrete mix. This process is critical for the development of strength and durability in concrete. The data presented in Figure 9 show that the degree of hydration increases with an increase in the water–cement ratio, as observed in [34,44,61,66,72,73,75,77,78,79,86,98,110,131]. This can be attributed to the availability of more water for the cement particles to initiate and sustain the hydration process. The observation that the maximum degree of hydration was achieved at a W/C ratio of 0.50 implies that the concrete mix had an optimal balance between water and cement, allowing for efficient hydration and strength development. Conversely, the lowest degree of hydration at a W/C ratio of 0.30 suggests that the limited water content hindered the hydration process, resulting in lower strength development.

Additionally, other factors, like aggregate type, admixtures, and curing conditions, also play significant roles in the overall performance of the concrete. Adjusting the W/C ratio within the specified range can help achieve the desired properties and performance of the concrete for various construction applications. Two types of calorimetry can be used to measure the heat generated during the hydration of cement: isothermal calorimetry and non-isothermal calorimetry. Isothermal calorimetry measures the heat generated at a constant temperature. It is useful for studying the kinetics of the hydration process and determining the reaction rates. Non-isothermal calorimetry measures the heat generated at varying temperatures. It is useful for studying the reaction hydration process’s thermodynamics and determining the reaction’s heat [60,97]. The heat generated during the hydration process can also be used to infer the rate of the hydration process, which can be useful for determining the curing time of concrete, determining the kinetics and thermodynamics of the hydration process, and determining the curing time of concrete [139].

### 2.14. Magnetic Sensor

Magnetic sensors can be used to measure the magnetic susceptibility of the cement as it hydrates. The change in magnetic susceptibility can be used to infer the degree of hydration. Magnetic sensors can be used in civil engineering for various applications, including measuring the position and movement of structures, such as bridges and buildings, to detect and monitor structural deformation and vibrations [140]; the detection of underground utilities, such as pipes and cables, during excavation and construction; the detection of metal objects, such as reinforcing steel, in concrete structures; measuring soil compaction and density during construction; monitoring the corrosion of metal structures, such as bridges and buildings; and the detection of metallic objects and mines in mining, constructions, and other similar industries [141,142].

**Figure 10:**
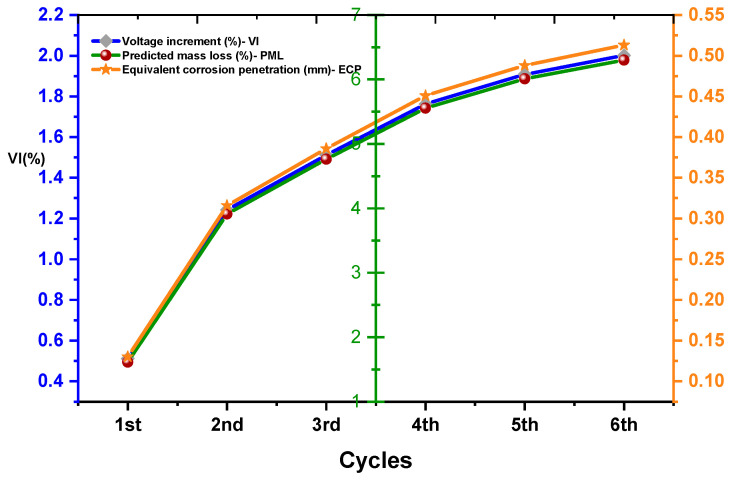
Covariation in voltage increment with equivalent corrosion penetration.

A magnetic sensor using this principle changes its electrical resistance in response to a magnetic field. The sensor typically consists of a thin film of a magneto-resistive material, such as giant magneto-resistive (GMR) material, that changes resistance in the presence of a magnetic field. The change in resistance is then converted into an electrical signal that can be measured and used to detect the presence or strength of the magnetic field [143]. The sensor typically consists of a thin semiconductor material, such as silicon or gallium arsenide, with a small voltage generated when a magnetic field is applied perpendicular to its surface. This voltage is known as the Hall voltage and can be used to detect the strength and direction of the magnetic field [144]. Both of the above principles are used in many applications, like detecting the position, orientation, and rotation of objects; measuring magnetic fields; and detecting the presence of metallic objects. Figure 10 shows the covariance of voltage increment with equivalent corrosion penetration. The covariance of voltage increment with equivalent corrosion penetration refers to the statistical relationship between the change in voltage and the amount of corrosion penetration that occurs in a material. The covariance of these variables can provide insights into the corrosion process and help us identify potential issues early on [145]. This information is essential in maintaining the safety and longevity of materials in various applications, such as in the construction industry, where corrosion can compromise the structural integrity of buildings and other structures [146]. The datasets obtained to plot Figure 10 obtained from the past literature [34,44,61,66,72,73,75,77,78,79,86,98,110,131].

These sensors measure a physical property that changes as the cement hydrates, such as temperature, wave velocity, resistance, heat, absorption, or magnetic susceptibility [37]. Table 2 shows crack detection, along with width and length measurements. Sensors like fiber-optic sensors and ultrasonic sensors play a vital role in detecting cracks in concrete. Fiber-optic sensors gauge changes in light transmission to determine crack width and length, enabling the timely identification and quantification of crack severity. Ultrasonic sensors, on the other hand, indirectly measure crack dimensions by analyzing changes in wave propagation. These data are invaluable, as they aid in assessing the extent of damage, guiding repair strategies, and preventing catastrophic failures.

Monitoring temperature with data, moisture, and corrosion considerations: Sensors, including fiber-optic and electrical resistivity sensors, facilitate continuous temperature monitoring within concrete structures. These data are essential for assessing the effects of temperature fluctuations on structural integrity. Additionally, these sensors can offer insights into moisture variations, which is crucial for preventing concrete degradation. Table 3 shows a comprehensive overview of technical specifications and sensor applications.

Many studies primarily focus on short-term monitoring. Future research could explore long-term performance of sensor systems and its durability in real-world structural environments to assess their reliability over extended periods. While individual sensors like fiber-optic sensors and ultrasonic sensors offer valuable insights, future research could focus on integrating multiple sensor technologies to enhance monitoring capabilities comprehensively. Integrating data from various sensors poses challenges in data processing and interpretation. Future research could explore advanced data fusion techniques to integrate and analyze data from diverse sensor types effectively. Some sensors may face challenges in harsh environmental conditions, such as extreme temperatures or high humidity. Future research could focus on developing sensor materials and designs that are robust and reliable under such conditions. While some sensor technologies mentioned may require wired connections, developing wireless monitoring systems could provide greater flexibility and scalability in structural health-monitoring applications. Many advanced sensor technologies can be expensive to implement, limiting their widespread adoption, particularly in smaller-scale projects. Addressing cost constraints and exploring cost-effective sensor solutions could broaden the applicability of structural health monitoring. Certain sensor technologies may require specialized expertise for installation and calibration, which can increase the complexity and cost of implementation. Simplifying installation procedures and developing user-friendly interfaces could mitigate this limitation. Interpreting data collected from sensors can be challenging, especially for non-specialists. Future research could focus on developing automated analysis algorithms and user-friendly interfaces to facilitate data interpretation and decision-making. Regular calibration and maintenance are crucial for ensuring the accuracy and reliability of sensor measurements. However, these tasks can be time-consuming and resource intensive. Future research could explore self-calibrating sensor designs and predictive maintenance techniques to minimize downtime and maintenance costs.

The sensor technologies discussed offer valuable insights into the structural health of concrete, enabling the early detection of defects and potential structural failures. They provide real-time monitoring capabilities, thus facilitating proactive maintenance and enhancing overall safety of concrete structures and its longevity. However, these technologies may have accuracy, reliability, and cost-effectiveness limitations. Additionally, the installation and data interpretation complexity can pose implementation challenges. Addressing these demerits through further research and development efforts is essential to maximize the potential benefits of structural health monitoring technologies in concrete structures.

## 3. Concrete Chronicles: A Sensor-Infused Structural Analysis

### 3.1. Interfacial Bond–Slip Model

**Figure 11:**
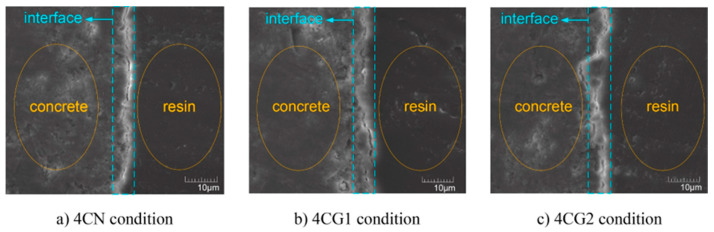
The microstructure of the interface between the sensor coating layer and the matrix concrete can be examined before loading (reproduced from [153]).

An interfacial bond–slip model is a mathematical representation of the behavior of the interface between two materials, such as a sensor and concrete. The model describes how the bond strength between the two materials changes as the interface is loaded and slips. Various types of bond–slip models have been proposed in the literature, each with its own assumptions and parameters [151]. One of the most commonly used models is the linear elastic–perfectly plastic model, which assumes that the bond strength is linear up to a certain point, after which it reaches a maximum value and stays constant. The slip is also assumed to be linear up to maximum bond strength [152]. Figure 11 offers a microstructure analysis of the sensor coating–matrix concrete interface before loading.

Another model is the bilinear model, which assumes that the bond strength is linear up to a certain point, after which it drops off rapidly. The slip is also assumed to be linear up to maximum bond strength. Another model is the trilinear model assumes that the bond strength increases initially, reaches a maximum value, and then drops off rapidly. The slip is also assumed to be linear up to maximum bond strength [154]. The choice of model and its parameters will depend on the specific application and the available experimental data. The models can be used to predict the behavior of interfaces under different loading conditions and to optimize the design of sensors and other structures [155].

The appearance of local failure on a specimen after fracture can provide valuable information about the behavior of the interface under different conditions. Typically, three main types of interface failure are observed. Cohesive failure occurs when the bond between the sensor and the matrix concrete is strong enough to resist the applied load. The failure occurs within the concrete, with little or no debonding at the interface. Adhesive failure occurs when the bond between the sensor and the matrix concrete is weaker than the concrete. The failure happens at the interface, with debonding and little or no cracking within the concrete. Mixed-mode failure occurs when the bond between the sensor and the matrix concrete has intermediate strength. The failure occurs both within the concrete and at the interface, with both debonding and cracking [156].

The specific failure appearance will depend on the properties of the sensor, the matrix concrete, and the specific conditions of the test. Understanding the failure appearance can help to identify the factors that control the bond strength and optimize the design of sensors and other structures [86].

The trend observed in the bond–slip behavior of a grooved, uncoated sensor versus a coated, grooved sensor can be understood step-by-step as the load increases. With an increase in load, the bond strength between the sensor and the matrix concrete also increases, leading to an increase in interfacial slip. However, the increase in bond strength and slip may not be linear; instead, the bond–slip curve may exhibit steps or plateaus, where the bond strength and slip remain constant for a certain range of loads before increasing again [157]. This type of behavior can occur due to the presence of grooves in the sensor surface and the use of acrylic coating. The grooves can create different slip planes, and the coating can act as a barrier, affecting the bond strength and slip behavior [158]. The specific details of the bond–slip curve, including the location and size of the steps or plateaus, will depend on the particular sensor, the matrix concrete, the acrylic coating, and the specific test conditions. Understanding the step-by-step trend in the bond–slip curve can provide insight into the factors that control the bond strength and slip and can help optimize the design of sensors and other structures [159].

If the peak load position in the bond–slip curve shows a sharp angle, the bond strength between the sensor and the matrix concrete is likely brittle, resulting in a rapid increase in interfacial slip with increased load. In such cases, a hyperbolic model may be a more suitable choice to describe the bond–slip behavior of the sensor. The hyperbolic model is commonly used to describe the behavior of brittle interfaces, such as concrete-to-steel or concrete-to-concrete interfaces [160]. This model assumes that the bond strength is proportional to the interfacial slip raised to a power, known as the “exponent” and represented by “n”. The higher the value of the exponent, the more brittle the interface behavior [161].

The sharp angle observed at the peak load position and the brittle behavior of the interface may suggest that a hyperbolic model could be used to describe the bond–slip behavior of the uncoated grooved sensor, coated grooved sensor, and matrix concrete. Utilizing a hyperbolic model could impedance rove the comprehension of the bond–slip behavior of the sensor and the matrix concrete in various conditions, thus aiding in the optimization of sensor and structure design [61].

### 3.2. Analytical Model of Embedded Corroded Steel Bar

An analytical model can be employed to predict the behavior of an embedded corroded steel bar in concrete under different loading conditions. The model involves solving equations that describe the mechanical properties of the steel and concrete and the impact of corrosion on the steel [62].

**Figure 12:**
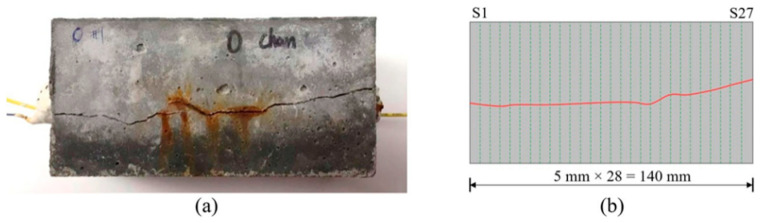
Tested beams characterized by crack width: (**a**) a beam with prevented longitudinal cracks, and (**b**) measurement sections (S1 to S27) show crack width (reproduced from [121]).

**Figure 13:**
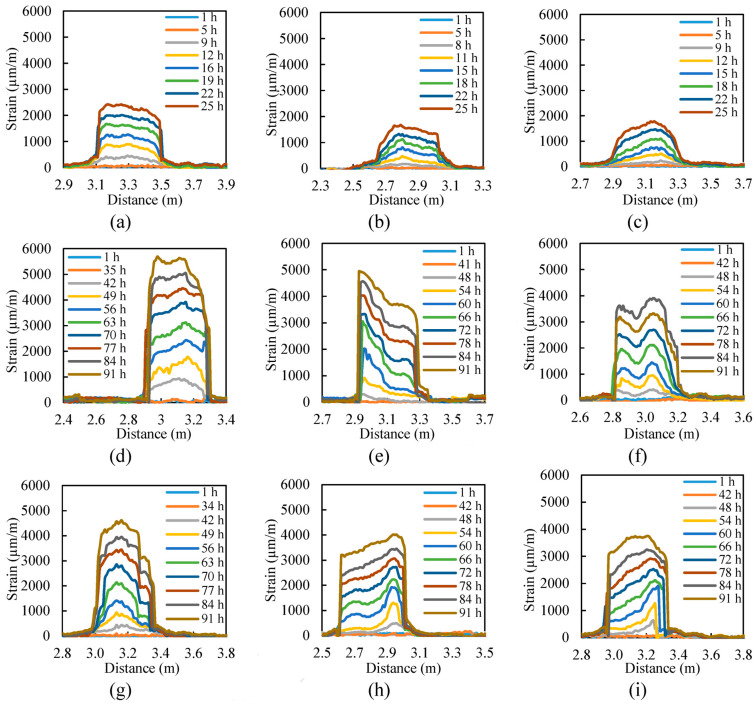
Strain distributions along distributed sensors in beams: (**a**) 0%-20, (**b**) 0.5%-20, (**c**) 1%-20, (**d**) 0%-10, (**e**) 0.5%-10, (**f**) 1%-10, (**g**) 0%-0, (**h**) 0.5%-0, and (**i**) 1%-0 (reproduced from [121]).

An effective method for modeling embedded corroded steel bars is using a bond–slip model. This model can accurately predict the behavior of the bond between the steel bar and concrete as the interfacial slip changes. The bond–slip model can also factor in the effects of corrosion on the bond strength and consider the mechanical properties of the steel and concrete. Additionally, a finite element model is another approach that can simulate the behavior of corroded steel bars and surrounding concrete in greater detail [63]. This model can consider the nonlinear behavior of steel and concrete and the complex geometries that can occur in corroded steel bars. Both models used to predict the behavior of embedded corroded steel bars under various loading conditions, such as tension, compression, and bending. The results can be used to understand the effects of corrosion on the steel bar and to design more durable and sustainable structures. It is worth mentioning that the analytical models can have some limitations and assumptions; it is important to validate the models with experimental data and when possible [120]. Figure 12 shows the characterization of tested beams based on crack width. The strain distributions along distributed sensors in beams are displayed in Figure 13.

A meso-scale model can be utilized to investigate the behavior of a steel–concrete composite with a fiber-optic cable instrumented at the steel–concrete interface under varying loading conditions. This model considers the mechanical characteristics of both the steel and concrete, along with the impact of the fiber-optic cable on the interface behavior [162]. Using a fiber-optic cable, interfacial strains and stresses can be measured between the steel and concrete, directly measuring bond–slip behavior at the steel–concrete interface. These data can help validate analytical models and better understand the behavior of the steel–concrete composite under various loading conditions [163].

A meso-scale model of a steel–concrete composite can be generated through various techniques, like the finite element method or the boundary element method. The model can incorporate the nonlinear behavior of the steel and concrete and account for the intricate geometries within the composite. The model can measure interfacial strains and stresses by installing fiber-optic cables at the steel–concrete interface, which facilitates direct measurement of the bond–slip behavior [164]. The results from the meso-scale model can shed light on the behavior of the steel–concrete composite under different loading conditions and improve the design and performance of steel–concrete structures. In addition, the fiber-optic cable can provide a more reliable and accurate measurement of the behavior of composite [79,165].

Faraday’s law states that the amount of charge (Q) that is transferred through an electrode during a redox reaction is directly proportional to the number of moles of electrons (n) that are transferred and can be calculated using the equation Q = nF, where F is the Faraday constant.

In the case of steel bars embedded in concrete beams undergoing corrosion, the amount of charge transferred during the corrosion reaction can be used to calculate the mass loss (Δm) of the steel bar. The number of moles of electrons transferred during the corrosion reaction of a steel bar can be calculated by assuming it to be a redox reaction and applying Faraday’s law. The mass loss of the steel bar can be calculated by multiplying the molar mass of steel by the number of moles of electrons transferred [140,166]. For example, in the case of 0–0%, 0–10%, and 0–20% beams, the mass loss can be calculated by measuring the amount of charge transferred during the corrosion reaction and applying Faraday’s Law, n = Q/F. Then, the mass loss can be calculated as Δm = n × molar mass of steel. It is worth mentioning that the mass loss determined from the charge transfer method is an approximate value, and it is important to validate the results with other methods, such as weight loss or electrochemical methods. The corrosion rate can also be determined from the mass loss and exposure time [84,93].

### 3.3. Fiber-Optic Sensors

**Figure 14:**
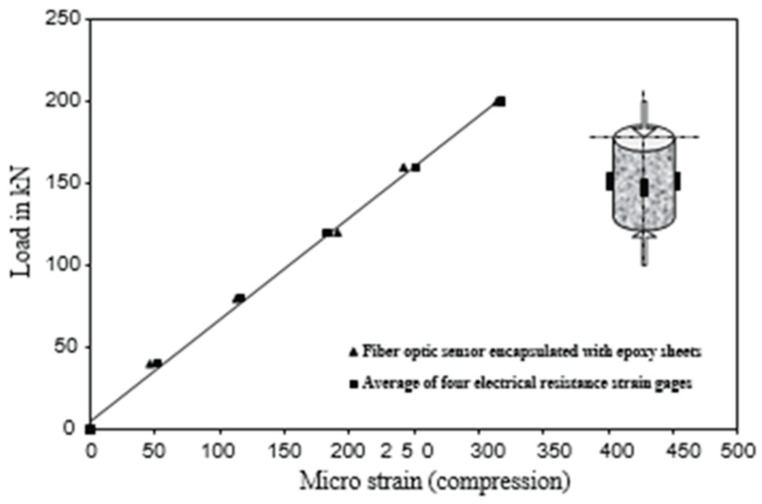
Comparison of strain response for fiber-optic sensor vs. electrical resistances strain gauge (reproduced from [122]).

During failure tests, the strain response of an epoxy-encapsulated fiber-optic sensor embedded in a structure can provide crucial information about the behavior of the sensor and the structure. The sensor can measure strains at its location during the test, which can offer valuable insight into the deformation and failure behavior of the structure [167]. A comparison of strain response between fiber-optic sensors and electrical resistance strain gauges can be seen in Figure 14.

During failure tests, the strain response of the sensor can be analyzed to understand the distribution and magnitude of strains in the structure at various stages of loading. The sensor can also detect and measure any changes in the strains that occur as the structure approaches or reaches failure [168].

The strain response of an epoxy-encapsulated fiber-optic sensor embedded in a structure can be evaluated using different techniques, such as wavelength-division multiplexing (WDM) or time-division multiplexing (TDM). These techniques enable the measurement of changes in the optical properties of the sensor, such as light intensity or phase. This information can then be used to determine the strains at the location of sensor, providing insight into the deformation and failure behavior of the structure [121].

The results of the strain response analysis can be used to better understand the structural behavior under different loading conditions and improve the design and performance of similar structures in the future. It can also help identify the location of the failure and e failure cause [169]. Concrete cylinders of grade M45 with embedded fiber-optic sensors can be used to study the behavior of concrete structures under various loading conditions. These cylinders can be prepared using different embedment techniques, such as the in situ or pre-casting embedment technique. In the in situ embedment technique, the fiber-optic sensors are embedded into the concrete mixture before it is poured into the cylindrical molds. This technique allows for the fiber-optic sensors to be placed in a specific location within the cylinder and can be useful for studying the behavior of the concrete at that specific location [123].

The pre-casting embedment technique embeds the fiber-optic sensors into a pre-cast concrete cylinder. This technique allows for the fiber-optic sensors to be placed at a specific location within the cylinder and can be useful for studying the behavior of the concrete at that specific location. Both types of embedment techniques have their advantages and disadvantages. The in situ embedment is more convenient for the study of the behavior of concrete at a specific location. Still, the process may be affected by the environment and the vibration during the casting process. The pre-casting embedment is more controllable but may not be able to study the behavior of the concrete at a specific location [124].

Once the concrete cylinders are prepared, they can be subjected to various loading conditions, such as compression, tension, and bending. The embedded fiber-optic sensors can measure the strains and deformation of the concrete at different points during the loading process. This can provide important information about the behavior of concrete and the structure performance under different loading conditions [125].

The temperature vs. strain plots for epoxy-encapsulated embedded sensors can be used to determine the effect of temperature on the strains measured by the sensors. The plots can be obtained by exposing the concrete cylinders with embedded sensors to different temperatures and measuring the strains at each temperature. The temperature correction coefficients can be obtained by analyzing the plots [126]. The correction coefficients can be used to adjust the strains measured by the sensors to account for the effect of temperature. These coefficients can be specific to the type of sensor, encapsulation material, and embedment technique used. It is noted that the difference between the two trials of temperature correction coefficient is less than 2%; this implies that the sensor and the encapsulation material are relatively insensitive to temperature changes, and the correction coefficients obtained from the trials are reliable [170]. It is also important to note that the temperature correction coefficients obtained from the experiments should be used cautiously as the real-world environments may have different temperature conditions than the test conditions.

### 3.4. Carbon Black and Polypropylene Fiber Subjected to Different Loading Conditions

The mechanical behavior of PP fiber/cementitious composites with varying dosages of CB (carbon black) under cyclic compression can be analyzed by studying their piezoresistive responses. PP fibers are polypropylene fibers commonly used as reinforcement in cement-based composites. Carbon black (CB) is a material added to the composite to improve its electrical conductivity.

When a piezoresistive sensor is embedded in the composite, it can measure the strain in the material under cyclic compression. The piezoresistive sensor changes its electrical resistance when subjected to strain; this change in resistance can be used to calculate the strain [127,171].

**Figure 15:**
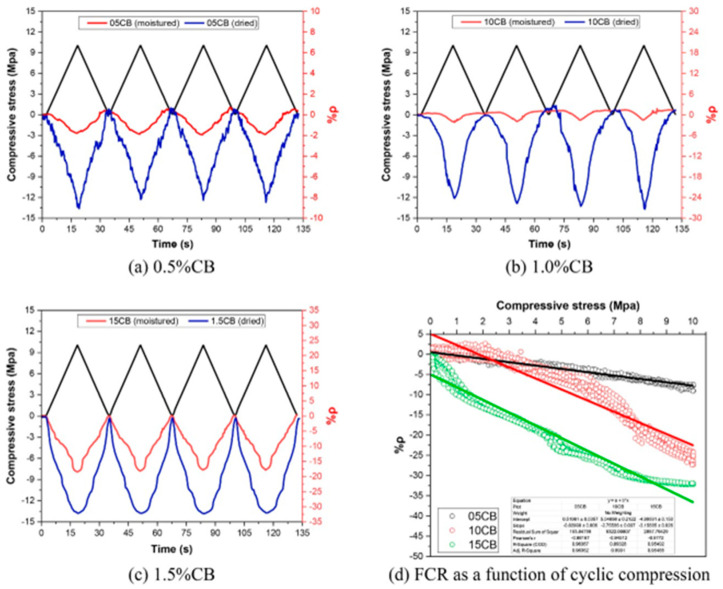
Piezo resistive responses of PP fiber/cementitious composites with varying CB dosages under cyclic compression (reproduced from [172]).

By studying the piezoresistive responses of the PP fiber/cementitious composites with different dosages of CB, it is possible to understand how the addition of CB affects the mechanical properties of the composite [64]. For instance, a higher dosage of CB can improve the electrical conductivity of composite and mechanical properties. The effect of varying CB dosages on the piezoresistive responses of PP fiber/cementitious composites under cyclic compression is illustrated in Figure 15.

It is important to note that the mechanical behavior of the composite will also be affected by other factors, such as the proportion and orientation of the PP fibers in the matrix, the curing conditions, and the type of cement used. Therefore, the results obtained from this study should be considered in the context of those variables. The cyclic compression of 10 MPa can be used to study the piezoresistive behavior of polypropylene (PP)-reinforced cementitious composites filled with different dosages of filler material in both undried and dried states. Piezoresistivity is the change in the electrical resistance of a material when it undergoes strain [64,173].

When a piezoresistive sensor is embedded in the composite, it can measure the strain in the material under cyclic compression. The sensor changes its electrical resistance when it is subjected to strain, which can be used to calculate the strain [174]. Studying the piezoresistivity of PP-reinforced cementitious composites filled with different dosages of filler material in both undried and dried states can help us understand how the mechanical properties of the composite are affected by the addition of the filler material and drying state. The piezoresistivity refers to the change in electrical resistance of the material when it is subjected to strain. For instance, a higher dosage of the filler material can improve the mechanical properties of the composite [128].

It is important to note that the mechanical behavior of the composite will also be affected by other factors, such as the proportion and orientation of the PP fibers in the matrix, the curing conditions, and the type of cement used. Therefore, the results obtained from this study should be considered in the context of those variables. Furthermore, it should be noted that the cyclic compression of 10 MPa is only one loading condition and may not represent the actual loading conditions experienced by the structure. Nonetheless, the study revealed that both the moisture and dry specimens exhibited distinctive piezoresistive behavior, indicating a change in the electrical resistance of the material when subjected to strain. Specifically, the resistivity of material decreased during loading and increased during unloading. This behavior is expected for piezoresistive materials, and it is consistent with the mechanical behavior of the material, as the material is undergoing compressive loading during the loading phase and tensile loading during the unloading phase.

It is important to note that the specific changes in resistivity will depend on the properties of the material and the loading conditions used in the experiment. This can be used to better understand mechanical behavior of the composite material and develop more accurate models for predicting its response under different loading conditions. Additionally, factors such as the proportion and orientation of the PP fibers, the type of cement used, and the curing conditions will also affect the mechanical properties of the composite.

### 3.5. Effect of Steel Fiber and Carbon Black on Concrete Cracks

Adding steel fibers and carbon black to concrete can enhance its self-sensing ability when subjected to bending. Steel fibers can improve toughness and ductility flexibility of the concrete, making it more resilient to cracking [175]. Additionally, including carbon black can help increase the electrical conductivity of the concrete, allowing for the more accurate sensing of cracks. When added in small amounts, carbon black can act as a conductive filler and improve the electrical conductivity of the concrete. This allows for a more accurate detection of cracks in the concrete using electrical resistance methods. Steel fibers and carbon black can enhance the self-sensing ability of concrete cracks by improving the toughness, ductility, and electrical conductivity of the concrete. The gauge factor measures how much the electrical resistance of a material changes in response to changes in strain. The addition of carbon materials and steel fibers can affect the gauge factor of concrete [175,176]. Carbon fibers can increase the gauge factor of concrete by improving its electrical conductivity. Carbon fibers are highly conductive, allowing for more accurate crack detection using electrical resistance methods [176].

**Figure 16:**
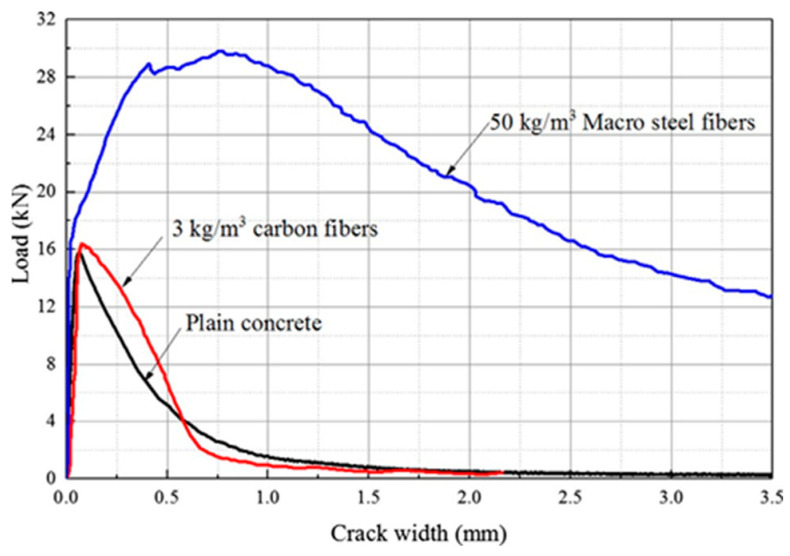
The load–COD (crack-opening displacement) relationship of PC, SF50, and CF03 (reproduced from) [177].

Steel fibers can also increase the gauge factor of concrete by improving its mechanical properties. Steel fibers can increase the toughness and flexibility of concrete, making it less prone to cracking. This can help reduce the gauge factor of concrete when loaded in bending. The load–COD (crack-opening displacement) relationship is depicted in Figure 16.

The addition of carbon materials and steel fibers can have a significant impact on the gauge factor of concrete. Carbon fibers can improve the electrical conductivity of concrete, thereby increasing the gauge factor. In contrast, steel fibers can enhance mechanical properties of concrete, thus decreasing the gauge factor. It should be noted that conventional concrete flexural members are designed to tolerate some cracking level during the service stage. As it is difficult to completely prevent cracking in concrete structures, monitoring the behavior of cracks is essential. However, conventional concrete with coarse aggregate has limited self-monitoring ability, as it lacks the added benefits of steel fibers and carbon black that can enhance the self-sensing ability of cracks [178,179,180]. Using steel fibers and carbon black in concrete can improve its self-monitoring ability by providing a more accurate way to detect and locate cracks. The steel fibers can improve the toughness and flexibility of the concrete, making it more resistant to cracking. At the same time, the carbon black can act as a conductive filler and improve the electrical conductivity of the concrete, allowing for a more accurate detection of cracks using electrical resistance methods. While it is impossible to eliminate cracking in concrete structures completely, adding steel fibers and carbon black can improve the self-monitoring ability of the concrete, making it more resistant to cracking and providing a more accurate way to detect and locate cracks. Carbon black and PP fibers have been used to improve the self-sensing capabilities of cementitious materials. The addition of carbon black nanoparticles as conductive fillers in cementitious composites has shown high-resolution strain measurement and stress-wave detection without external instruments [1]. Incorporating electrostatic self-assembly carbon nanotube–nano carbon black (CNT-NCB) composite fillers (CNCFs) in glass fiber reinforced polymer (GFRP) reinforced concrete beams has achieved low and stable electrical resistivity, allowing for in situ monitoring of strain and damage accumulation [131]. The distribution of conductive phases and the coating efficiency of carbon black nanoparticles on the surfaces of PP fibers heavily influence the self-sensing performance of PP fiber cement-based sensors, providing strain- and deformation-sensing and crack- and damage-detection capabilities [65,129,130,177,178,179,180,181,182,183,184,185,186,187,188,189,190,191,192,193,194,195,196,197,198,199,200,201]. Additionally, using carbon black-filled cement-based sensors mixed with silicone hydrophobic powder (SHP) and crystalline waterproofing admixture (CWA) has shown improved water impermeability and chloride resistance, making them suitable for structural health monitoring applications. Incorporating carbon fibers in cementitious mixes, particularly sea components, has also improved the electrical conductivity and sensitivity of the materials, making them suitable for smart-city infrastructures.

Carbon black nanoparticles were added to cementitious composites to enhance their self-sensing capacity for low-amplitude strain. The piezo-resistivities of the composites with carbon black nanoparticles were recorded during low-amplitude cyclic loadings. The electrical resistance variance between two closely contacted electrodes was collected as a signal for strain measurement and compared with signals from lead zirconate titanate (PZT) sensors. The developed materials showed the potential for high-resolution strain measurement and stress wave detection without external instruments [178,179,180].

### 3.6. Nile Blue-Immobilized pH Sensor Monitors the Concrete Carbonation

Concrete carbonation is a chemical reaction where carbon dioxide from the air reacts with the calcium hydroxide in concrete, leading to a reduction in pH and a consequent decrease in the strength of the concrete. This process can lead to the degradation of structures over time, making timely maintenance and monitoring important for ensuring the safety and serviceability of the structures. The sensor described was made by covalent immobilization, in which Nile blue, a dye that changes color in response to pH, was chemically bonded to cellulose, a natural polymer. This technique allows the sensor to be stable and retain its sensitivity over time. Additionally, the reaction process for the sensor is relatively simple and short, making it easy to use and convenient for monitoring concrete carbonation. Destructive methods of measuring concrete carbonation involve taking a concrete sample and analyzing the pore pH of solution or concrete powder suspension. This is performed by dissolving the chemicals present in the concrete and allowing them to reach an equilibrium of dissolution. These methods involve destroying a small part of the concrete structure. Still, they provide an accurate measurement of the carbonation level, which helps determine the integrity of the structure and the need for maintenance or repair.

**Figure 17:**
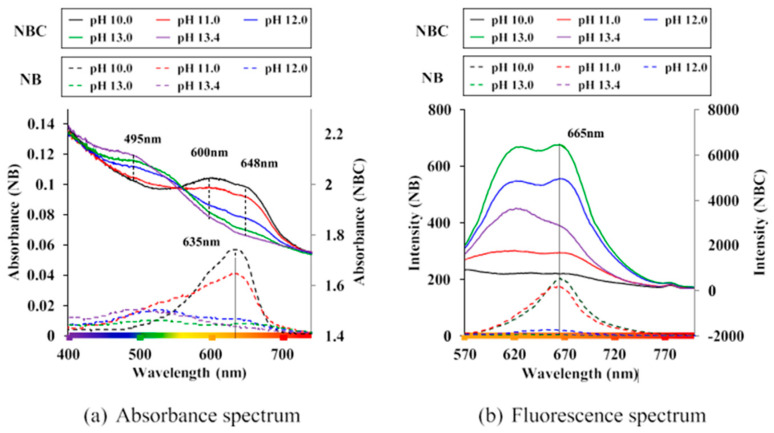
The absorption and fluorescence spectrum for NB and NCB (reported from [85]).

A pH sensor that uses trinitrobenzene sulfonic acid (TNBS) and methyltriethoxysilane (MTEOS) to monitor concrete carbonation by entrapping TNBS into MTEOS. A sensor that changes color in response to pH changes can be used to monitor the carbonation of concrete over time (refer to Figure 17). This method is non-destructive and can be used to continuously monitor the pH level of concrete in real time, providing a reliable and accurate picture of the carbonation level and the need for maintenance.

A comparison of the FTIR (Fourier-Transform Infrared Spectroscopy) results between Nile blue-immobilized cellulose and alpha-cellulose can be used to verify the covalent bonding between Nile blue and alpha-cellulose. FTIR is a technique that measures the absorption of infrared radiation by a sample and can be used to identify the functional groups present in a compound. When Nile blue is covalently bonded to cellulose, the FTIR spectrum should show characteristic peaks for the Nile blue molecule and the cellulose. In particular, the FTIR spectrum of Nile blue-immobilized cellulose should show peaks for the amine (-NH_2_) and carboxylic acid (-COOH) groups of the Nile blue molecule and peaks for the cellulose.

The comparison between the FTIR spectrum of Nile blue-immobilized cellulose and alpha-cellulose can be used to verify that Nile blue is covalently bonded to the cellulose. If the FTIR spectrum of Nile blue-immobilized cellulose shows the same peaks as the FTIR spectrum of alpha-cellulose, likely the Nile blue is not covalently bonded to the cellulose. However, if the FTIR spectrum of Nile blue-immobilized cellulose shows additional peaks that are not present in the FTIR spectrum of alpha-cellulose, it is likely that Nile blue is covalently bonded to the cellulose. An accelerated carbonation test is a method used to evaluate the resistance of concrete to carbonation, which is a process that occurs when carbon dioxide in the air reacts with the calcium hydroxide in concrete, resulting in the formation of calcium carbonate. The test involves exposing a concrete sample to a high concentration of carbon dioxide in a controlled environment, such as a chamber, to simulate the carbonation process over a short period of time.

A sensor system that can monitor the early stage of concrete carbonation would be able to detect changes in the pH and/or the electrical conductivity of the concrete, as these are indicators of carbonation. For instance, as carbonation occurs, the pH of concrete drops and electrical conductivity increases. The applicability of the sensor system can be verified by comparing the results obtained from the sensor system with those obtained from conventional methods of measuring carbonation, such as measuring the depth of carbonation using a drill or measuring the pH of the concrete using a pH meter. The sensor system should be able to provide similar or comparable results to the conventional methods, and it should be able to detect the early stages of carbonation in the concrete. The sensor system could also monitor the concrete carbonation over time and detect any changes in the carbonation rate.

### 3.7. Embedded Piezoelectric Sensor for Crack Analysis in Concrete

**Figure 18:**
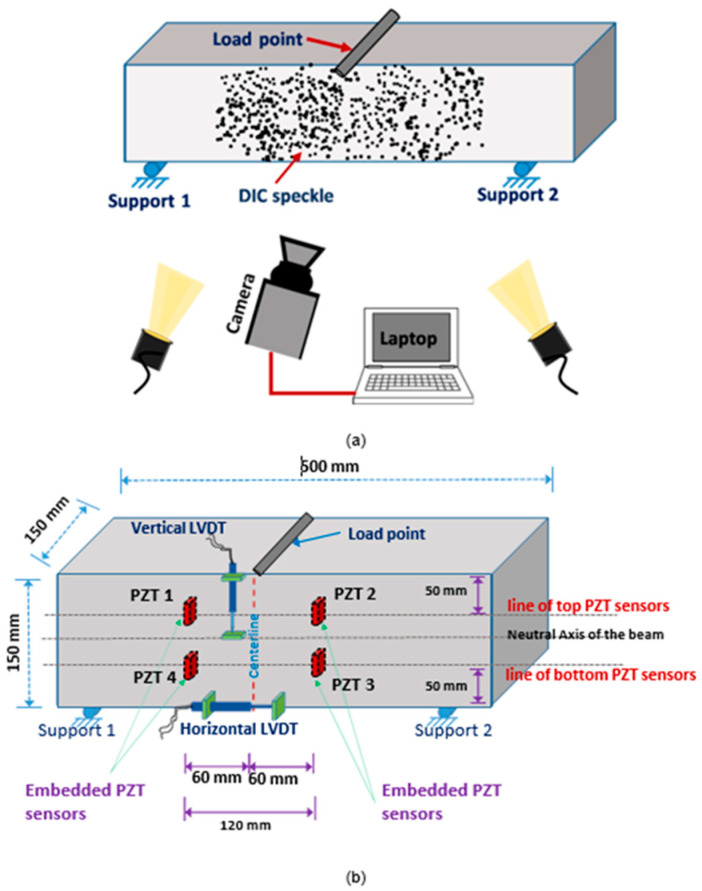
(**a**,**b**) Schematic representation of embedded PZT sensor in beam (reproduced from [183]).

**Figure 19:**
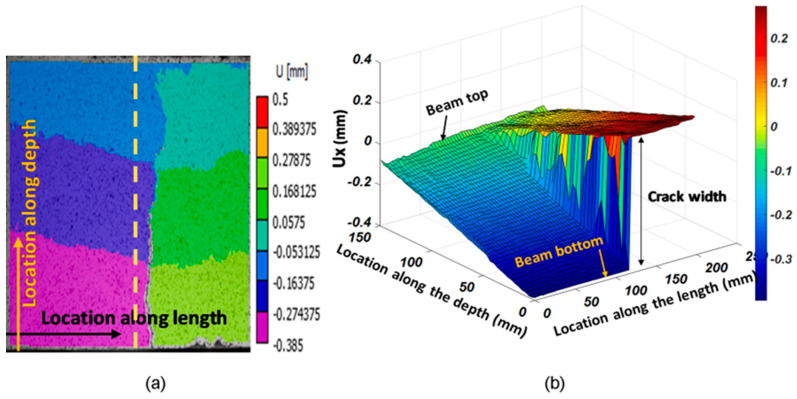
(**a**,**b**) The displacement of the horizontal surface of a concrete beam in the L400 state can be represented using 2D and 3D contour plots (reproduced from) [183].

**Figure 20:**
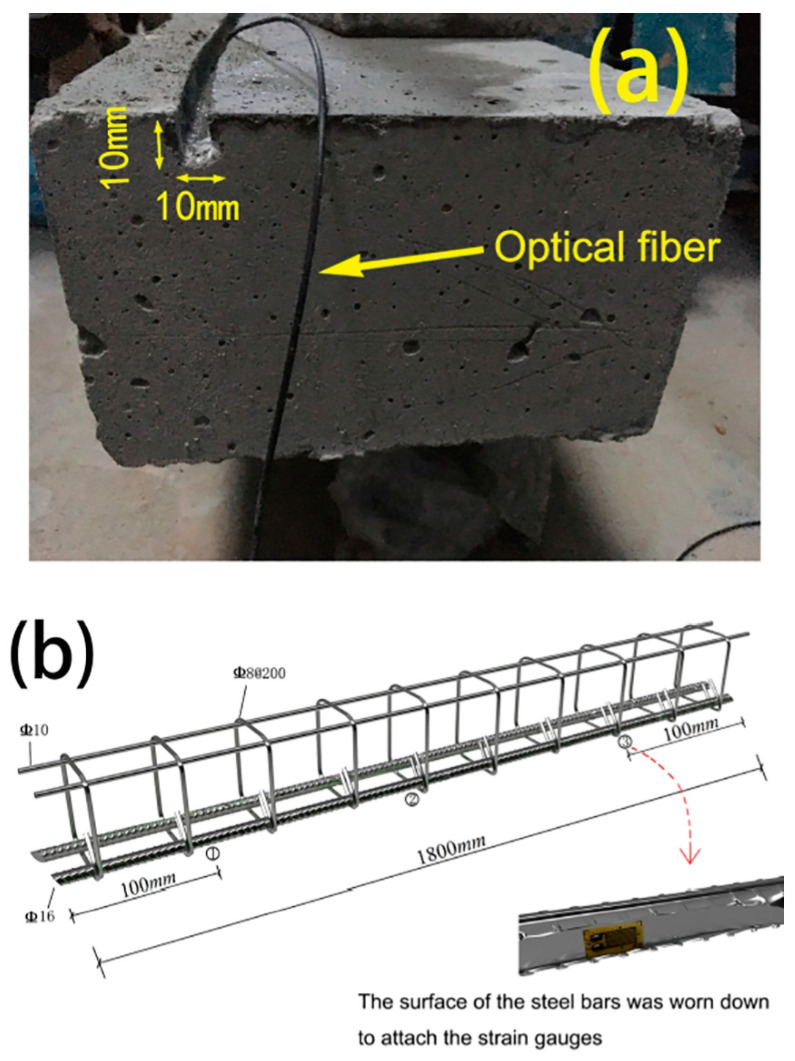
Optical fiber and strain gauge layout: (**a**) embedding optical fibers in concrete beams and (**b**) positioning strain gauges in the steel cage (reproduced from [91]).

Stress-wave measurements are a technique used to study the mechanical properties of materials, including concrete. Stress waves are elastic waves that travel through a material in response to an applied force [42]. By measuring the speed and attenuation of stress waves, it is possible to infer information about the microstructure of material, strength, and damage state. Stress-wave measurements can be employed to investigate the different stages of damage localization that lead to the formation, propagation, and opening of cracks in concrete [181]. This can be achieved by applying a known load to a concrete sample and measuring the resulting stress waves traveling through the material. By analyzing the stress-wave data, it is also possible to detect changes in the mechanical properties of the concrete that occur as cracks form, propagate, and open [182]. Figure 18 shows a schematic representation of an embedded PZT sensor in a beam, which detects structural changes and monitors the structural health. The displacement of the surface of the concrete beam is shown in the contour plot and Optical fiber and strain gauge layout in concrete beam are in Figure 19 and Figure 20.

During the crack formation, the stress-wave measurements detect the elastic waves generated by the microcracks that appear in the concrete that are not visible to the human eye. As the crack propagates, the stress-wave measurements detect the waves that travel through the crack and reflect off the crack surfaces, allowing us to estimate the crack width. When the crack opens, stress-wave measurements detect the changes in the amplitude of the stress waves, as the amplitude is a function of the crack width and the distance between the crack surfaces [38]. These measurements can estimate the crack opening displacement (COD). This method of stress-wave measurements is non-destructive and can be used to study the damage progression in a concrete structure over time. This is useful in assessing the long-term durability of the structure and determining when maintenance or repairs may be needed. Conductance spectroscopy is a technique used to study the electrical properties of materials, including hardened concrete [65]. In this technique, a small electrical current is applied to a material, and the resulting electrical conductance is measured as a function of frequency. The conductance spectrum is a plot of the conductance as a function of frequency, and it can provide information about the microstructure, composition, and defects in the material [184].

When PZT sensors are embedded in hardened concrete and a small electrical current is applied, the resulting conductance spectrum can provide information about the state of the concrete. PZT sensors are piezoelectric materials that generate an electrical charge in response to applied stress or strain. By measuring the electrical conductance of the concrete, it is possible to infer information about the stress and strain state of the material [183]. The conductance spectrum of hardened concrete will depend on the microstructure, composition, and defects; conductance will increase with frequency, as the electrical current encounters fewer defects and interfaces. The presence of cracks, pores, and voids in the concrete could also affect the conductance spectrum, decreasing the conductance at lower frequencies. The conductance spectrum can also be used to detect changes in the mechanical properties of the concrete over time [185]. For instance, if the conductance decreases at lower frequencies, it could indicate that the concrete is cracking and losing strength. Conductance increases at lower frequencies; it could indicate that the concrete is healing and gaining strength.

A three-point loading configuration is a test setup in which a beam or other structural element is supported at two points, and a load is applied at a third point. This is a common configuration used to test the behavior of structures beams flexural strength and deflection characteristics, and behavior of the structures under bending. In this configuration, the load is applied perpendicular to the longitudinal axis of the beam at a specific point, and the displacement and deformation of the beam are measured at different locations [129,187]. Digital Image Correlation (DIC) is a non-destructive technique that enables the measurement of deformation and displacement of structures. The technique involves applying speckle patterns on the surface and correlating the patterns of structure. In a DIC setup, a camera captures images of the speckle pattern on the structure surface before and after loading. The displacement and deformation of the structure at different points can be determined by comparing the two images. DIC is commonly used with a three-point loading configuration, where the speckle pattern is applied to the front face of the beam, and the camera captures images of the speckle pattern before and after loading [128,188]. The DIC software then compares the two images to determine the displacement and deformation of the beam at different points. This combination of techniques allows for the measurement of the displacement and deformation of the beam under loading, providing detailed information about the flexural behavior of the beam and the distribution of stresses and strains within the material. It can also be used to study the behavior of structures under different loading conditions, such as fatigue and impact loading [189,190].

### 3.8. Optical Fiber Sensor in Concrete Testing for Strain Transfer Model

Distributed optical fibers for structural health monitoring involve embedding the optical fibers within the structure, enabling continuous monitoring of the structure’s mechanical properties, such as strain, temperature, and vibration [191]. However, one of the primary challenges associated with this approach is ensuring the survival of the optical fibers. These fibers are susceptible to damage from mechanical loads, environmental conditions, and installation processes [192]. The optical fibers are typically inserted into the structure for protection to ensure their survival.

There are several ways to protect the optical fibers in a structure:

- Embedding the optical fibers within a protective coating can include protective coatings such as epoxy or polyurethane. These coatings protect mechanical loads and environmental conditions.
- Inserting the optical fibers within a protective tubing: This can include metal or plastic tubing designed to protect the optical fibers from mechanical loads and environmental conditions.
- Incorporating the optical fibers into the structure: This can include embedding the optical fibers within the structure, such as within the concrete or other building materials.
- Using Fiber Bragg Grating (FBG) sensors: FBG sensors are written directly onto the optical fiber, thus providing a more robust and durable sensor for harsh environments.

Protecting the optical fibers in this way makes it possible to ensure their survival rate and the integrity of the structural health monitoring system. There are several methods of arranging optical fibers and strain gauges for structural health monitoring using distributed optical fibers [193]. One method involves embedding the optical fibers into concrete beams. One way to lay out the optical fibers is to embed them within the concrete beams of the structure. This can be performed by running the optical fibers through the center of the concrete beams or along the surface of the beams. The optical fibers can be embedded in a protective coating or tubing to ensure their survival rate. Another approach for laying out the optical fibers and strain gauges is to position them within a steel cage embedded within the structure [194]. The strain gauges are attached to the steel cage at specific points in this configuration. The optical fibers are then run along the surface of the steel cage and are connected to the strain gauges. This allows for the monitoring of the strain on the steel cage, which can be used to infer the strain on the surrounding concrete [195].

These layouts are commonly used to monitor the structural integrity of the concrete beams and steel cage. The layout chosen will depend on the specific requirements of the structure and the type of data that need to be collected. The temperature calibration-test results of the tight-sheath strain-sensing fiber refer to the measurements taken to evaluate the accuracy and performance of the fiber-optic sensor system under different temperature conditions [196]. The goal of the test is to determine how the output of the sensor system changes as the temperature of the environment changes. During the test, the tight sheath strain sensing fiber is placed in a controlled environment where the temperature varies. The output of the sensor system is then measured at various temperatures. The data collected from the test are then analyzed to determine how the sensor system output changes with temperature [197]. The results of the temperature calibration test can be presented in the form of a graph, showing the relationship between the sensor system output and the environmental temperature. The results can be used to determine the sensor system accuracy, identify any temperature-related errors, and make any necessary adjustments to the sensor system to improve its performance. It is important to note that the tight-sheath strain-sensing fiber is a fiber-optic sensor that uses a tight buffer coating around the optical fiber to measure the strain applied on the fiber in real time [130]. This type of sensor is commonly used in harsh environments and where high accuracy is needed. The temperature calibration test was performed using a new type of tight sheath strain sensing optical fiber developed to enhance the accuracy and performance of strain monitoring. This type of fiber is based on an ordinary strain optical fiber, but with some modifications that make it more suitable for monitoring strain in specific applications [198].

The tight-sheath strain-sensing fiber is designed to have a tight buffer coating around the optical fiber. This tight buffer coating is designed to protect the optical fiber from external factors such as temperature, humidity, and mechanical stress, which can affect the accuracy of the sensor system. Additionally, it helps to increase the sensitivity of sensor system, allowing for more accurate strain measurements [199]. The tight-sheath strain-sensing fiber has a wide range of applications, including monitoring the structural integrity of infrastructure such as bridges and buildings, and in industrial settings, where high accuracy and durability are required. It can also be used in harsh environments, such as high temperatures and high humidity, where other strain-sensing fibers may not be suitable [200]. This type of fiber is an improvement over the traditional strain-sensing fibers. It is more sensitive and durable and can withstand harsh environmental conditions. It is a good option for applications requiring high accuracy and long-term monitoring [201].

## 4. Factors Influencing the Sensor-Based Study in Concrete

**Figure 21:**
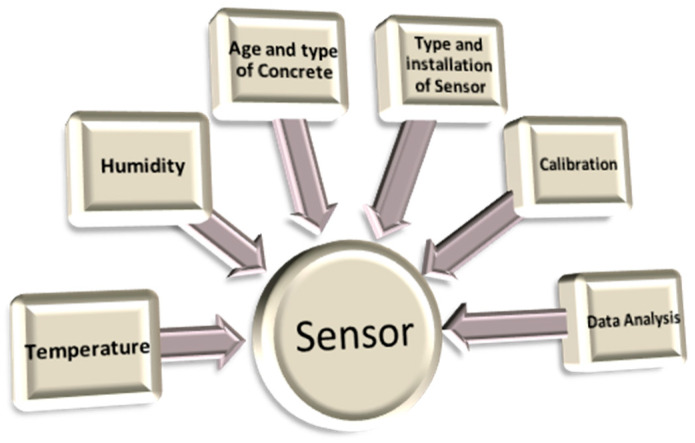
Factors influencing sensor-based studies in concrete.

Several factors can influence the accuracy and reliability of sensor-based studies in concrete (refer to Figure 21). Temperature changes can cause expansion and contraction in the concrete, which can affect the accuracy of sensor measurements. High humidity levels can cause the concrete to absorb moisture, leading to changes in its mechanical properties, which can affect sensor readings.

As concrete ages, it changes its mechanical properties, which can affect sensor readings. Different types of concrete, such as normal-weight or lightweight concrete, can have different properties that can affect sensor readings. The type of sensor used, such as strain gauges or fiber-optic sensors, can also influence the accuracy of sensor-based studies in concrete [202]. The installation of the sensor can also affect the accuracy of the sensor-based study, such as the location and orientation of the sensor, which can affect the accuracy of the sensor readings. Proper sensor calibration is important in ensuring the accuracy of sensor-based studies in concrete. How the data are analyzed can also affect the accuracy of sensor-based studies in concrete. Considering these factors and taking appropriate measures to mitigate their effects when performing sensor-based studies in concrete [203].

## 5. Sensor-Based Studies in Concrete

**Figure 22:**
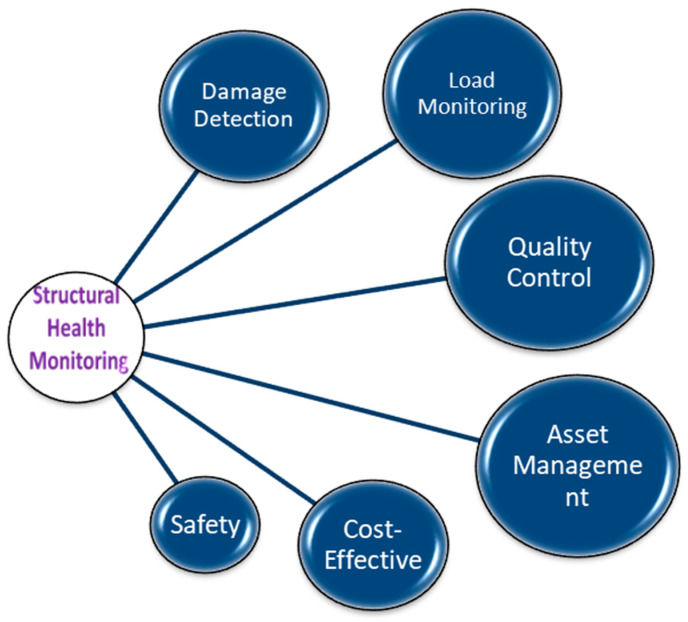
Structural health monitoring and its aspects.

Structural health monitoring: Sensors can monitor the structural integrity of concrete structures, such as bridges, buildings, and other infrastructure (refer to Figure 22). This allows engineers to identify potential problems before they become critical and to take appropriate measures to prevent failure [204]. Sensors can detect damage in concrete structures, such as cracks, delamination, and other forms of deterioration. This allows engineers to identify and repair problems before they lead to structural failure. Sensors can be used to monitor the loads and stresses on concrete structures, allowing engineers to optimize the structure design and ensure that it can withstand the loads it is subjected to. Sensors can be used to monitor the quality of the concrete during the construction process, ensuring that the concrete is poured and cured correctly and meets the necessary strength and durability requirements [205,206,207]. Sensors can monitor the condition of concrete structures over time, providing valuable data that can be used to plan maintenance and repair work and extend the service life of a structure. A sensor-based study is a cost-effective way to monitor the condition of a structure; it can help to identify potential problems early on, preventing major repairs and costs in the future. Sensor-based studies can ensure safety by detecting the potential failure of a structure, thus providing an early warning and avoiding the risk of collapse. Sensor-based studies in concrete are important because they allow engineers to understand structural behavior of concrete better and take appropriate measures to ensure its safety and longevity [208].

## 6. Vibrational-Based Monitor in Concrete Structure

**Figure 23:**
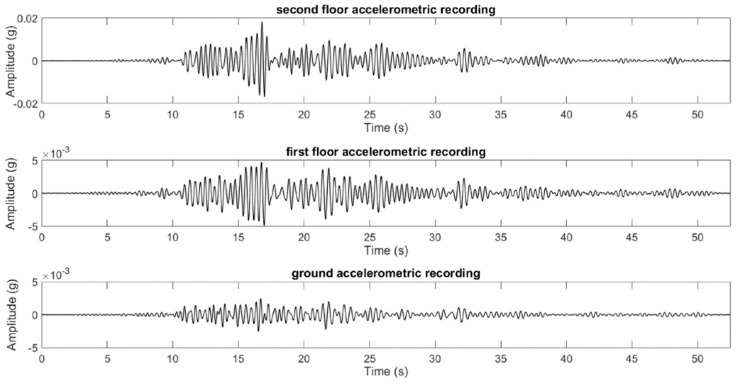
Accelerometer readings were taken at various floors of the Navelli municipal building in the transverse direction (Reproduced from [102]).

Structural health monitoring has gained interest due to the limitations of traditional methods. Automatic systems offer the potential for rapid and effective assessments of structures. The continuous estimation of structural modal properties can detect changes due to material deterioration and earthquake damage. The “Curvature Evolution Method” is a technique for detecting and localizing damage in framed structures. It involves tracking the evolution of curvature within the structure to identify areas where damage may be present. This method has been modified and validated through numerical simulations and experimental studies. Researchers conducted case studies using numerical models and physical experiments to validate the modified approach. These studies aimed to demonstrate the effectiveness and reliability of the CEM in accurately detecting and localizing damage within framed structures. Researchers have defined empirical relationships between the variation in curvature and a damage index. This relationship allows for a quantitative assessment of damage severity based on changes in curvature. Importantly, this relationship has been established for both bare frames and frames with infill, providing a versatile tool for assessing structural health across different framed structures. The Navelli town hall experienced significant damage during the earthquake on 6 April 2009, with a magnitude of 6.3. Starting from 7 April 2009, ambient noise measurements were conducted on the structure, and by 8 April, a local accelerometric network was installed both outside and inside the building. Regarding nonstructural elements, substantial damage was observed in both the external infill panels and internal partitions. The infill panels exhibited extensive cracking at the ground and first stories along both the longitudinal and transverse frames. The areas near the stiff stair structure and along the longitudinal direction were particularly affected. Wide cracks were found along the longitudinal direction of the infill panels in the last bay of the ground and first story. Damage along the transverse direction was less severe, primarily manifesting as in-plane cracking. Diagonal cracking in the panels, resulting from inclined tension stress concentrated in the central region, and corner crushing due to interaction with the surrounding frame were notable damage mechanisms observed. The structural response acceleration at each floor was initially analyzed to implement the damage detection method [102]. Figure 23 illustrates an example of the accelerometric recordings corresponding to the event recorded on 9 April 2009, at 04:32 a.m.

Machine learning algorithms are extensively used in structural health monitoring (SHM) for detecting damage in various civil engineering structures. ML algorithms are classified into vibration-based SHM and image-based SHM, showcasing their efficacy in analyzing clustering, regression, and damage classification. Deep learning, a subset of ML that utilizes neural networks with multiple layers, has gained significant attention in SHM. Convolutional Neural Networks (CNNs) have shown promise in image-based SHM tasks, such as crack detection in concrete structures or corrosion assessment in steel bridges. ML algorithms are increasingly integrated with advanced sensor technologies, such as accelerometers, strain gauges, and drones equipped with cameras or LiDAR. These sensors provide rich data streams that ML algorithms can analyze to detect and characterize damage in real time. There is a growing trend towards fusing data from multiple sources, including vibration, temperature, strain, and visual data, to improve the accuracy and robustness of damage detection algorithms. ML techniques are being developed to handle multimodal data fusion, enabling comprehensive structural health assessment. Unsupervised learning techniques, such as clustering and anomaly-detection algorithms, are being explored for detecting subtle changes or anomalies in structural behavior that may indicate damage. These techniques are particularly useful for continuously monitoring structures where labeled training data may be scarce. Transfer learning, where knowledge gained from training ML models on one task is transferred to another related task, is gaining traction in SHM. Pre-trained models, especially those trained on large-scale datasets from other domains like computer vision, can be fine-tuned for specific SHM tasks, reducing the need for extensive labeled data. ML algorithms capable of online learning and adaptation are being developed for SHM applications. These systems can continuously update their models based on incoming data, allowing them to adapt to changing environmental conditions and damage patterns in real time. As ML algorithms become more complex, there is a growing emphasis on developing explainable AI techniques that provide insights into how models make decisions. Interpretable models enhance trust and understanding, especially in safety-critical applications like SHM [109].

## 7. Some of the Many Challenges Associated with Using Sensors for Monitoring Concrete Structures

- Sensor placement: Proper sensor placement is critical for accurately monitoring the structure. If the sensors are not placed in the right location, they may not be able to detect important changes in the structure condition. The sensors must be placed in locations that detect critical changes in the condition of the structure. This includes areas where the structure is most likely to experience stress, such as at points of high loading or where cracks are likely to form. Additionally, sensors should be placed at different depths within the structure to detect changes at different levels. The sensors should be placed in a way that allows for the most complete coverage of the structure so that any changes can be detected as early as possible [209].
- Sensor calibration: Sensors must properly calibrate to produce accurate and reliable data. This can be a time-consuming and costly process, and it may need to be repeated periodically to ensure the accuracy of the sensor readings. Calibration includes adjusting the sensitivity of the sensor, offset, and other parameters to ensure it reads correctly. The calibration process can be performed in a laboratory, before the sensor is installed; or it can be performed on-site, after the sensor has been installed. Sometimes, sensors may need to be recalibrated periodically to ensure that they continue producing accurate measurements over time. There are different methods of calibrating sensors, depending on the type of sensor. For example, some sensors may be calibrated using a known reference point, while others may be calibrated using a series of measurements taken at different points. The specific calibration method used will depend on the sensor and the application for which it is being used. It is important to note that if the sensor is not calibrated correctly, it could lead to inaccurate results, which can have serious consequences for the safety and performance of the monitored structure [194,210].
- Sensor durability: Concrete structures are often exposed to harsh environmental conditions, such as high temperatures, humidity, and vibration. Sensors must withstand these conditions to provide accurate and reliable data over time. These factors can cause a sensor to degrade or malfunction, resulting in inaccurate or unreliable data. Some sensors are designed to be more rugged and withstand harsh conditions than others. For example, sensors that are designed for use in outdoor environments may be more weather-resistant than sensors that are designed for use indoors. Similarly, sensors designed for use in high-vibration environments may be more shock-resistant than those designed in low-vibration environments [211].

Additionally, if a sensor is exposed to high levels of humidity or temperature, the output of the sensor will be affected by it, thus affecting the results. Hence, the sensor should be specifically designed to withstand those conditions. When selecting sensors for a particular application, it is important to consider the environmental conditions to which the sensors will be exposed and choose sensors that can withstand them. This will help ensure that the sensors provide accurate and reliable data over time.

- Sensor interference: Some sensors may be affected by other sources of electromagnetic interference, which can cause inaccuracies in the sensor readings.
- Data analysis: The data collected by sensors must be analyzed to extract useful information. This process can be complex and time-consuming, requiring specialized software and expertise.
- Data storage and transmission: Collecting, storing, and transmitting large amounts of sensor data can also be challenging.
- Cost: Sensors can be expensive and may need to be replaced or recalibrated periodically, which can add to the cost of monitoring a concrete structure.
- Maintenance: Proper care and maintenance of the sensors are necessary for their longevity and accuracy.

The challenges associated with using sensors for monitoring concrete structures include proper sensor placement, calibration, durability, and data analysis, which can require specialized knowledge and resources.

## 8. Future Scope of Sensor-Based Study in Concrete

The future scope of sensor-based study in concrete is wide and promising. With advancements in sensor technology and data analysis, it is becoming increasingly possible to monitor the condition of concrete structures in real time and predict their behavior over time. There are key areas where sensor-based studies in concrete are expected to have a significant impact in the future. For instance, sensors can be used to detect early signs of damage in concrete structures, such as cracks or changes in stiffness. This can help engineers identify potential problems before they become critical, allowing them to take proactive measures to prevent failure [194,196,212,213].

Non-destructive testing: Sensors can perform non-destructive testing (NDT) on concrete structures, which can help engineers evaluate the structure condition without causing damage. Sensors can be used to create “smart” concrete structures that can automatically adjust their behavior in response to changes in the environment or their condition. Sensors can be used to evaluate the long-term durability of concrete structures, which can help engineers optimize the design and construction of new structures to improve their longevity. Sensors can be used to monitor the quality of concrete during construction, which can help ensure that the concrete meets the required specifications [72,214]. Sensor-based study in concrete can revolutionize how we design, construct, and maintain concrete structures, making them safer, more efficient, and more sustainable. Sensor-based geopolymer concrete has been the subject of significant research efforts, but some areas remain where further investigation is needed. One of these is sensor integration, where more research is required on how different types of sensors can be combined to provide more accurate and reliable data on the properties of geopolymer concrete [215,216,217].

The development of automatically self-sensing concrete is currently a research hotspot in construction materials. This innovative concrete is designed to possess the ability to detect and respond to changes in its environment without requiring external sensors or monitoring devices. Self-sensing concrete typically incorporates conductive materials, such as carbon nanotubes, carbon fibers, or metallic particles, into the concrete mix. These materials enable the concrete to conduct electricity and react to various stimuli, such as changes in strain, stress, temperature, or moisture [218,219,220,221,222]. One of the primary objectives of self-sensing concrete is to enhance structural health monitoring in buildings, bridges, and other infrastructure. By integrating self-sensing capabilities directly into the concrete itself, engineers and maintenance personnel can obtain real-time data on the condition and performance of the structure, enabling timely maintenance and repair interventions. The potential applications of self-sensing concrete are vast, ranging from the early detection of structural defects and damage to monitoring the effectiveness of structural reinforcements and repairs. Additionally, self-sensing concrete has the potential to contribute to the development of smart cities and infrastructure by enabling more efficient and cost-effective maintenance strategies. Addressing these research gaps will help to advance the use of sensor-based geopolymer concrete further and promote its wider application in the construction industry [223,224].

## 9. Conclusions

This review article is a significant contributor to several UN SDGs. It pioneers sensor technologies for concrete monitoring (Goal 9), enhancing safety in urban areas (Goal 11) and resilience to climate change (Goal 13). It also aids in coastal preservation (Goal 14) and habitat protection (Goal 15) through effective monitoring techniques. With advances in sensor technology and data analysis, monitoring concrete structures in real time and predicting their behavior over time is now possible.

- The analysis with “actual”, “fits”, and “trending line” variables, using MAPE, MAD, and MSD: MAPE (5.54857) showed the average percentage difference between predicted and actual values. MAD (1.64708) represented the average deviation from the mean, highlighting dataset variability. MSD (4.40477) indicated the average magnitude of errors, measuring the squared differences between predicted and actual values.
- For water-to-cement (W/C) ratios of 0.3, 0.4, and 0.5, the shear strength values ranged between 4.4 and 5.6 MPa. The varying W/C ratios directly affect the workability of concrete and compactness, leading to different strengths. A W/C ratio of 0.4 demonstrated the highest shear strength, likely due to the optimal balance between water content and cement, resulting in improved particle packing and hydration. For the W/C ratio of 0.3, although slightly lower than the optimal 0.4, the shear strength remained relatively high, indicating effective particle packing and hydration, leading to a solid intermolecular structure.
- A higher water–cement ratio means more water for cement to react, increasing hydration. Optimal hydration at W/C 0.50 shows the ideal balance for strong concrete. Lower hydration at W/C 0.30 implies limited water hampers strength development. An embedded PZT sensor in a beam detects structural changes and monitors its health. A contour plot shows the surface displacement of the concrete beam. Stress-wave measurements capture microcracks forming in the concrete, invisible to the naked eye. These measurements track wave propagation and reflection off crack surfaces, aiding in estimating crack width.
- Adding steel fibers and carbon black to concrete improves its ability to sense changes when bent. Carbon fibers enhance the electrical conductivity of concrete, thus aiding accurate crack detection. Similarly, steel fibers boost the gauge factor by improving mechanical properties and reducing susceptibility to cracking. This ultimately decreases the gauge factor under bending loads, as indicated by the load–COD (crack-opening displacement).
- Establishing a quantitative relationship between curvature changes and damage severity provides a versatile tool for assessing structural health in various framed structures. Extensive cracking was observed in the infill panels, particularly near the stiff stair structure and along the longitudinal direction. Wide cracks were evident in the last bay of the ground and first story, with less severe damage along the transverse direction, mainly manifested as in-plane cracking. Diagonal cracking and corner crushing were notable damage mechanisms, highlighting the complex interaction between tension stress and the surrounding frame.
- These sensors help us to understand the early warning signs in systems with non-destructive testing, smart structures, durability assessment, and quality control. These advancements will help engineers optimize the design and construction of new structures and help ensure the safety and longevity of existing structures. The future of sensor-based study in concrete is promising, and it is expected to impact the field of civil engineering significantly.

## Figures and Tables

**Table 1:** Techniques for structural health monitoring (SHM).

Technique	Physical Principle	Main Sensor Type	Range	Ref.
Acoustic emission	Detects and measures ultrasonic waves generated by cracks and other damage	Microphone, piezoelectric sensor	Depends on the material, size, and shape of the structure and the type of damage	[4,33,34,35,36]
Vibration analysis	Measures changes in frequency, amplitude, and mode shapes of vibrating structures.	Accelerometer	Typically, 0 to 20 KHz	[37,38]
Infrared thermography	Measures temperature changes in structures	Infrared camera	Typically −20 °C to 1500 °C	[39,40]
Guided waves	Propagates elastic waves along a structure to detect changes in its properties	Piezoelectric transducer, laser Doppler vibrometer	Typically, 0.1 to 2 MHz	[4,41]
Optical fiber sensing	Uses changes in light transmission through optical fibers to detect changes in structural properties	Optical fiber, optical spectrum analyzer	Typically, from a few centimeters to several meters	[42,43]
Electromagnetic methods	Measures changes in electrical conductivity, magnetic permeability, and electromagnetic field	Eddy current probe, magnetic field sensors	Typically, 0 to 100 KHz	[44,45]
Cement/concrete-based sensors	Piezoresistivity refers to the property of a material to change its electrical resistance when subjected to mechanical stress or strain.	Piezoresistivity	Depends on the specific material and its composition, as well as the magnitude and direction of the applied stress	[46]

**Table 2:** Comparison of various sensors on different parameters.

Sensor Type	Crack Detection	Strain and Deformation Analysis	Temperature Monitoring	Moisture Monitoring	Corrosion Monitoring	Ref.
Fiber-optic sensors	↑↑↑	↑	↑	↑	↑↑↑	[34,53]
Strain gauges	↑↑↑	↑↑↑	↑	↑	↑↑↑	[2,3]
Acoustic emission sensors	↑	↑	↑	↑	↑	[54,55,100,106]
Ultrasonic sensors	↑↑↑	↑↑	↑↑	↑	↑	[6,33,107]
Electrical resistivity sensors	↑	↑	↑	↑	↑↑↑	[67,69,70,71,72,73,76,147,148]

↑—low; ↑↑—medium; ↑↑↑—high.

**Table 3:** Insights into sensor technology: specifications and practical uses.

Sensor Type	Technical Specifications	Applications	Ref.
Accelerometer	✓ Measures acceleration and vibration ✓ Range: Typically, ±2 g to ±200 g ✓ Frequency Response: 0 Hz to 2 kHz ✓ Output: Analog or digital	✓ Structural health monitoring ✓ Impact detection ✓ Seismic activity detection	[10,23,31,33,34,35,36,39,40,41,42,43,44]
Strain Gauge	✓ Measures strain in materials under stress ✓ Used in bridges, buildings, and dams ✓ Output: Electrical resistance change ✓ Gauge Factor: 2 to 6	✓ Load and stress analysis ✓ Structural integrity assessment ✓ Weight and pressure measurement	[47,59,73,74,80,82]
Inclinometer	✓ Measures inclination or slope ✓ Range: ±1 to ±90 degrees ✓ Output: Analog or digital ✓ Used for tilt monitoring in structures	✓ Slope stability monitoring ✓ Monitoring of retaining walls and slopes ✓ Foundation settlement detection	[15,18,20,23,24,25,29,30,76,147,148,149]
Moisture Sensor	✓ Measures moisture content in materials Detection Range: 0% to 100% moisture ✓ Output: Analog or digital ✓ Used in soil, concrete, and wood	✓ Concrete curing monitoring ✓ Waterproofing assessment ✓ Preventing mold and decay in wood structures	[56,57,58,59,72,77,150]
Thermocouple	✓ Measures temperature through voltage Temperature Range: −200 °C to 2000 °C ✓ Output: Voltage or temperature difference used in harsh environments and high temperatures	✓ Concrete curing temperature monitoring ✓ HVAC system monitoring ✓ Fire detection in buildings	[87,88,89,90,91,92,95]

## References

[1] Cao Y., Li J., Sha A., Liu Z., Zhang F., Li X. (2022). A power-intensive piezoelectric energy harvester with efficient load utilization for road energy collection: Design, testing, and application. J. Clean. Prod..

[2] Hassan A., Arif M., Shariq M. (2020). A review of properties and behaviour of reinforced geopolymer concrete structural elements—A clean technology option for sustainable development. J. Clean. Prod..

[3] Chiriatti L., Mercado-Mendoza H., Apedo K.L., Fond C., Feugeas F. (2019). A study of bond between steel rebar and concrete under a friction-based approach. Cem. Concr. Res..

[4] Meng Q., Wu C., Su Y., Li J., Liu J., Pang J. (2019). A study of steel wire mesh reinforced high performance geopolymer concrete slabs under blast loading. J. Clean. Prod..

[5] Yue L., Yang Y., Zhou Q., Lei Y., Deng G., Yang T. (2022). Broadband electromagnetic wave absorbing performance by designing the foam structure and double-layer for cement-based composites containing MWCNTs. Cem. Concr. Compos..

[6] Materazzi A.L., Ubertini F., D’Alessandro A. (2013). Carbon nanotube cement-based transducers for dynamic sensing of strain. Cem. Concr. Compos..

[7] Khan M.I., Fares G., Abbas Y.M. (2022). Cost-performance balance and new image analysis technique for ultra-high performance hybrid nano-based fiber-reinforced concrete. Constr. Build. Mater..

[8] Park Z.-T., Choi Y.-S., Kim J.-G., Chung L. (2005). Development of a galvanic sensor system for detecting the corrosion damage of the steel embedded in concrete structure: Part 2. Laboratory electrochemical testing of sensors in concrete. Cem. Concr. Res..

[9] Gülşan M.E., Alzeebaree R., Rasheed A.A., Niş A., Kurtoğlu A.E. (2019). Development of fly ash/slag based self-compacting geopolymer concrete using nano-silica and steel fiber. Constr. Build. Mater..

[10] Mandal R., Chakraborty S., Chakraborty P., Chakraborty S. (2019). Development of the electrolyzed water based set accelerated greener cement paste. Mater. Lett..

[11] Afzal J., Yihong Z., Afzal U., Aslam M. (2023). A complex wireless sensors model (CWSM) for real time monitoring of dam temperature. Heliyon.

[12] Choi W.-C., Yun H.-D., Cho C.-G., Feo L. (2014). Attempts to apply high performance fiber-reinforced cement composite (HPFRCC) to infrastructures in South Korea. Compos. Struct..

[13] Shi L., Lin S.T.K., Lu Y., Ye L., Zhang Y.X. (2018). Artificial neural network based mechanical and electrical property prediction of engineered cementitious composites. Constr. Build. Mater..

[14] Arezoumandi M., Volz J.S. (2013). Effect of fly ash replacement level on the shear strength of high-volume fly ash concrete beams. J. Clean. Prod..

[15] Wu J., Jing H., Gao Y., Meng Q., Yin Q., Du Y. (2022). Effects of carbon nanotube dosage and aggregate size distribution on mechanical property and microstructure of cemented rockfill. Cem. Concr. Compos..

[16] Han X., Li G., Wang P., Chen Z., Cui D., Zhang H., Tian L., Zhou X., Jin Z., Zhao T. (2022). A new method and device for detecting rebars in concrete based on capacitance. Measurement.

[17] Tarbozagh A.S., Rezaifar O., Gholhaki M., Abavisani I. (2020). Magnetic enhancement of carbon nanotube concrete compressive behavior. Constr. Build. Mater..

[18] Jongvivatsakul P., Thongchom C., Mathuros A., Prasertsri T., Adamu M., Orasutthikul S., Lenwari A., Charainpanitkul T. (2022). Enhancing bonding behavior between carbon fiber-reinforced polymer plates and concrete using carbon nanotube reinforced epoxy composites. Case Stud. Constr. Mater..

[19] Javahershenas F., Gilani M.S., Hajforoush M. (2021). Effect of magnetic field exposure time on mechanical and microstructure properties of steel fiber-reinforced concrete (SFRC). J. Build. Eng..

[20] Naji H.F., Khalid N.N., Alsaraj W.K., Habouh M.I., Marchetty S. (2021). Experimental investigation of flexural enhancement of RC beams with multi-walled carbon nanotubes. Case Stud. Constr. Mater..

[21] Li X., Qin Z., Zheng D., Zhang X., Li H. (2023). Reversed bond-slip model of deformed bar embedded in concrete based on ensemble learning algorithm. J. Build. Eng..

[22] Pang Y., Li Z., Wang Q., Qi B. (2023). Effects of the liquid rubber modified adhesive on the bond-slip response of the CFRP-steel interface. J. Build. Eng..

[23] Liu J., Xie X., Li L. (2022). Experimental study on mechanical properties and durability of grafted nano-SiO_2_ modified rice straw fiber reinforced concrete. Constr. Build. Mater..

[24] Liu Z., Lu Y., Li S., Zong S., Yi S. (2020). Flexural behavior of steel fiber reinforced self-stressing recycled aggregate concrete-filled steel tube. J. Clean. Prod..

[25] Metaxa Z.S., Seo J.-W.T., Konsta-Gdoutos M.S., Hersam M.C., Shah S.P. (2012). Highly concentrated carbon nanotube admixture for nano-fiber reinforced cementitious materials. Cem. Concr. Compos..

[26] Xu T., Bian X., Liu Z., Yang J., Zhang Z. (2023). Local bond stress–slip relationship of ribbed reinforcing bars embedded in UHPC: Experiment, modeling, and verification. J. Build. Eng..

[27] Hou H., Wang W., Chen Y. (2023). Cyclic behavior and mechanical model of a novel endplate connection to double-skin composite wall with slip-critical blind bolts. J. Build. Eng..

[28] de Alencar Monteiro V.M., Cardoso D.C.T., de Andrade Silva F. (2023). Mechanisms of fiber-matrix interface degradation under fatigue loading in steel FRC. J. Build. Eng..

[29] D’Alessandro A., Tiecco M., Meoni A., Ubertini F. (2021). Improved strain sensing properties of cement-based sensors through enhanced carbon nanotube dispersion. Cem. Concr. Compos..

[30] Gupta S., Lin Y.-A., Lee H.-J., Buscheck J., Wu R., Lynch J.P., Garg N., Loh K.J. (2021). In situ crack mapping of large-scale self-sensing concrete pavements using electrical resistance tomography. Cem. Concr. Compos..

[31] Lu D., Shi X., Zhong J. (2022). Interfacial bonding between graphene oxide coated carbon nanotube fiber and cement paste matrix. Cem. Concr. Compos..

[32] Wang L., Song Y., Han Q., Wang Z. (2023). Experimental investigation on the dynamic behavior of grouted splice sleeve connector under fast tensile loading. J. Build. Eng..

[33] Dong W., Li W., Wang K., Han B., Sheng D., Shah S.P. (2020). Investigation on physicochemical and piezoresistive properties of smart MWCNT/cementitious composite exposed to elevated temperatures. Cem. Concr. Compos..

[34] Pourzahedi L., Zhai P., Isaacs J.A., Eckelman M.J. (2017). Life cycle energy benefits of carbon nanotubes for electromagnetic interference (EMI) shielding applications. J. Clean. Prod..

[35] Karayannis C.G., Chalioris C.E., Angeli G.M., Papadopoulos N.A., Favvata M.J., Providakis C.P. (2016). Experimental damage evaluation of reinforced concrete steel bars using piezoelectric sensors. Constr. Build. Mater..

[36] Wang X., Hu S., Li W., Qi H., Xue X. (2021). Use of numerical methods for identifying the number of wire breaks in prestressed concrete cylinder pipe by piezoelectric sensing technology. Constr. Build. Mater..

[37] Frankowski P.K., Chady T., Zieliński A. (2021). Magnetic force induced vibration evaluation (M5) method for frequency analysis of rebar-debonding in reinforced concrete. Measurement.

[38] Feng Q., Liang Y. (2022). Development of piezoelectric-based technology for application in civil structural health monitoring. Earthq. Res. Adv..

[39] Xu D., Banerjee S., Wang Y., Huang S., Cheng X. (2015). Temperature and loading effects of embedded smart piezoelectric sensor for health monitoring of concrete structures. Constr. Build. Mater..

[40] Ai D., Du L., Li H., Zhu H. (2022). Corrosion damage identification for reinforced concrete beam using embedded piezoelectric transducer: Numerical simulation. Measurement.

[41] Zhang H., Li J., Kang F., Zhang J. (2022). Monitoring and evaluation of the repair quality of concrete cracks using piezoelectric smart aggregates. Constr. Build. Mater..

[42] Li K., Li Y., Dong P., Wang Z., Dou T., Ning J., Dong X., Si Z. (2022). Pressure test of a prestressed concrete cylinder pipe using distributed fiber optic sensors: Instrumentation and results. Eng. Struct..

[43] Bai H., Guo D., Wang W., Tan X., Yan M., Chen G., Bao Y. (2022). Experimental investigation on flexural behavior of steel-concrete composite floor slabs with distributed fiber optic sensors. J. Build. Eng..

[44] Li K., Li Y., Dong P., Wang Z., Dou T., Ning J., Dong X., Si Z., Wang J. (2022). Mechanical properties of prestressed concrete cylinder pipe with broken wires using distributed fiber optic sensors. Eng. Fail. Anal..

[45] Fan L., Bao Y. (2021). Review of fiber optic sensors for corrosion monitoring in reinforced concrete. Cem. Concr. Compos..

[46] Dong W., Li W., Guo Y., Qu F., Wang K., Sheng D. (2022). Piezoresistive performance of hydrophobic cement-based sensors under moisture and chloride-rich environments. Cem. Concr. Compos..

[47] Dong X., Dou T., Dong P., Wang Z., Li Y., Ning J., Wei J., Li K., Cheng B. (2022). Failure experiment and calculation model for prestressed concrete cylinder pipe under three-edge bearing test using distributed fiber optic sensors. Tunn. Undergr. Space Technol..

[48] Tan X., Abu-Obeidah A., Bao Y., Nassif H., Nasreddine W. (2021). Measurement and visualization of strains and cracks in CFRP post-tensioned fiber reinforced concrete beams using distributed fiber optic sensors. Autom. Constr..

[49] Kim D., Kim R., Min J., Choi H. (2023). Initial freeze–thaw damage detection in concrete using two-dimensional non-contact ultrasonic sensors. Constr. Build. Mater..

[50] Wolf J., Pirskawetz S., Zang A. (2015). Detection of crack propagation in concrete with embedded ultrasonic sensors. Eng. Fract. Mech..

[51] Yuan L., Zhou L., Jin W. (2004). Long-gauge length embedded fiber optic ultrasonic sensor for large-scale concrete structures. Opt. Laser Technol..

[52] Song H., Feldman S.B., Popovics J.S. (2022). In situ detection and characterization of alkali-silica reaction damage in concrete using contactless ultrasonic wavefield imaging. Cem. Concr. Compos..

[53] Lefever G., Charkieh A.S., Abbass M., Van Hemelrijck D., Snoeck D., Aggelis D.G. (2023). Ultrasonic evaluation of self-healing cementitious materials with superabsorbent polymers: Mortar vs. concrete. Dev. Built Environ..

[54] Cheng W., Fan Z., Tan K.H. (2023). Characterisation of corrosion-induced crack in concrete using ultrasonic diffuse coda wave. Ultrasonics.

[55] Ge L., Li Q., Wang Z., Li Q., Lu C., Dong D., Wang H. (2023). High-resolution ultrasonic imaging technology for the damage of concrete structures based on total focusing method. Comput. Electr. Eng..

[56] Lee S.-J., Ahn D., You I., Yoo D.-Y., Kang Y.-S. (2020). Wireless cement-based sensor for self-monitoring of railway concrete infrastructures. Autom. Constr..

[57] Abner M., Wong P.K.-Y., Cheng J.C.P. (2022). Battery lifespan enhancement strategies for edge computing-enabled wireless Bluetooth mesh sensor network for structural health monitoring. Autom. Constr..

[58] Li J., Sun G., Wang A., Lei M., Liang S., Kang H., Liu Y. (2022). A many-objective optimization charging scheme for wireless rechargeable sensor networks via mobile charging vehicles. Comput. Netw..

[59] Siringoringo D.M., Fujino Y., Suzuki M. (2023). Long-term continuous seismic monitoring of multi-span highway bridge and evaluation of bearing condition by wireless sensor network. Eng. Struct..

[60] Janků M., Cikrle P., Grošek J., Anton O., Stryk J. (2019). Comparison of infrared thermography, ground-penetrating radar and ultrasonic pulse echo for detecting delaminations in concrete bridges. Constr. Build. Mater..

[61] Zhao H., Li J., Wang R., Lam D., Zhang Y. (2021). Study on interfacial bond behavior of recycled aggregate concrete filled stainless steel tubes (RAC-FSST). Constr. Build. Mater..

[62] Song J., Wang W., Su S., Wang B., Li Y., Lu Z. (2021). Experimental investigation of the bond-slip behaviour between corrugated steel plates and concrete in CSRC structures. Constr. Build. Mater..

[63] Ahmed K.S., Shahjalal M., Siddique T.A., Keng A.K. (2021). Bond strength of post-installed high strength deformed rebar in concrete. Case Stud. Constr. Mater..

[64] Jiang S.-F., Wang J., Tong S.-Y., Ma S.-L., Tuo M.-B., Li W.-J. (2021). Damage monitoring of concrete laminated interface using piezoelectric-based smart aggregate. Eng. Struct..

[65] Bansal T., Talakokula V., Sathujoda P. (2022). Durability aspects of blended concrete systems subjected to combined mechanical and environmental loading using piezo sensor. Constr. Build. Mater..

[66] Ramani V., Kuang K.S.C. (2021). Monitoring of rebar corrosion in concrete structures using a lens-based plastic optical fiber (LPOF) sensor. Constr. Build. Mater..

[67] Ramani V., Zhang L., Kuang K.S.C. (2021). Probabilistic assessment of time to cracking of concrete cover due to corrosion using semantic segmentation of imaging probe sensor data. Autom. Constr..

[68] Bansal T., Talakokula V., Saravanan T.J. (2022). Monitoring of prestressed concrete beam under corrosion using embedded piezo sensor based on electro-mechanical impedance technique. Sci. Talks.

[69] Ahmadi J., Feirahi M.H., Farahmand-Tabar S., Fard A.H.K. (2021). A novel approach for non-destructive EMI-based corrosion monitoring of concrete-embedded reinforcements using multi-orientation piezoelectric sensors. Constr. Build. Mater..

[70] Fu C., Huang J., Dong Z., Yan W., Gu X.-L. (2020). Experimental and numerical study of an electromagnetic sensor for non-destructive evaluation of steel corrosion in concrete. Sens. Actuators A Phys..

[71] Li Z., Jin Z., Gao Y., Zhao T., Wang P., Li Z. (2020). Coupled application of innovative electromagnetic sensors and digital image correlation technique to monitor corrosion process of reinforced bars in concrete. Cem. Concr. Compos..

[72] Chang C.-Y., Hung S.-S. (2012). Implementing RFIC and sensor technology to measure temperature and humidity inside concrete structures. Constr. Build. Mater..

[73] Mahapatra C.K., Barai S.V. (2019). Temperature impact on residual properties of self-compacting based hybrid fiber reinforced concrete with fly ash and colloidal nano silica. Constr. Build. Mater..

[74] Huang B., Wang J., Piukovics G., Zabihi N., Ye J., Saafi M., Ye J. (2023). Hybrid cement composite-based sensor for in-situ chloride monitoring in concrete structures. Sens. Actuators B Chem..

[75] Du Z., Wang P., Chen Z., Cui D., Jin Z., Zhang H. (2022). All-solid-state; long term stable, and embedded pH sensor for corrosion monitoring of concrete. J. Build. Eng..

[76] Li Z., Jin Z., Xu X., Zhao T., Wang P., Li Z. (2020). Combined application of novel electromagnetic sensors and acoustic emission apparatus to monitor corrosion process of reinforced bars in concrete. Constr. Build. Mater..

[77] Kim J., Luis R., Smith M.S., Figueroa J.A., Malocha D.C., Nam B.H. (2015). Concrete temperature monitoring using passive wireless surface acoustic wave sensor system. Sens. Actuators A Phys..

[78] Zou X., Chao A., Tian Y., Wu N., Zhang H., Yu T.-Y., Wang X. (2012). An experimental study on the concrete hydration process using Fabry–Perot fiber optic temperature sensors. Measurement.

[79] Górriz B.T., Payá-Zaforteza I., García P.A.C., Maicas S.S. (2017). New fiber optic sensor for monitoring temperatures in concrete structures during fires. Sens. Actuators A Phys..

[80] Wong A.C.L., Childs P.A., Berndt R., Macken T., Peng G.-D., Gowripalan N. (2007). Simultaneous measurement of shrinkage and temperature of reactive powder concrete at early-age using fibre Bragg grating sensors. Cem. Concr. Compos..

[81] Dong W., Li W., Tao Z., Wang K. (2019). Piezoresistive properties of cement-based sensors: Review and perspective. Constr. Build. Mater..

[82] Norris A., Saafi M., Romine P. (2008). Temperature and moisture monitoring in concrete structures using embedded nanotechnology/microelectromechanical systems (MEMS) sensors. Constr. Build. Mater..

[83] Gaibor N., Mateus R., Leitão D., Cristelo N., Miranda T., Pereira E.N.B., Cunha V.M.C.F. (2023). Sustainability assessment of half-sandwich panels based on alkali-activated ceramic/slag wastes cement versus conventional building solutions. J. Clean. Prod..

[84] Yuan L., Jin W., Zhou L., Lau K. (2001). The temperature characteristic of fiber-optic pre-embedded concrete bar sensor. Sens. Actuators A Phys..

[85] Jeong H., Jung B.J., Kim J.H., Seo S.-Y., Yun H., Kim K.S. (2022). Development and assessment of Nile blue-immobilized pH sensor to monitor the early stage of concrete carbonation. J. Build. Eng..

[86] Qasim M., Lee C.K., Zhang Y.X. (2022). An experimental study on interfacial bond strength between hybrid engineered cementitious composite and concrete. Constr. Build. Mater..

[87] Fernandez I., Berrocal C.G., Rempling R. (2023). Two-dimensional strain field analysis of reinforced concrete D-regions based on distributed optical fibre sensors. Eng. Struct..

[88] Yang Z., Chen Q., Li X., Chen H., Wang Z., Huang R., Kong Q. (2022). Crack identification in concrete structures using implantable sensors. Measurement.

[89] Singh I., Dev N., Pal S. (2022). Impedance based damage assessment of concrete under the combined effect of impact and temperature using different piezo configurations. Sens. Actuators A Phys..

[90] Ai D., Yang Z., Li H., Zhu H. (2021). Heating-time effect on electromechanical admittance of surface-bonded PZT sensor for concrete structural monitoring. Measurement.

[91] Li Z., Hou G., Hu T., Zhou T., Xiao H. (2021). Study on establishing and testing for strain transfer model of distributed optical fiber sensor in concrete structures. Opt. Fiber Technol..

[92] Zheng Z., Ji H., Zhang Y., Cai J., Mo C. (2022). High-entropy (Ca_0.5_Ce_0.5_)(Nb_0.25_Ta_0.25_Mo_0.25_W_0.25_)O_4_ scheelite ceramics with high-temperature negative temperature coefficient (NTC) property for thermistor materials. Solid State Ion..

[93] Fulham-Lebrasseur R., Sorelli L., Conciatori D. (2022). Prefabricated electrically conductive concrete (ECC) slabs with optimized electrode configuration and integrated sensor system. Cold Reg. Sci. Technol..

[94] Compaoré A., Sawadogo M., Sawadogo Y., Ouedraogo M., Sorgho B., Seynou M., Blanchart P., Zerbo L. (2023). Preparation and characterization of foamed concrete using a foaming agent and local mineral resources from Burkina Faso. Results Mater..

[95] Zuo Z., Huang Y., Pan X., Zhan Y., Zhang L., Li X., Zhu M., Zhang L., De Corte W. (2021). Experimental research on remote real-time monitoring of concrete strength for highrise building machine during construction. Measurement.

[96] Keo S.A., Brachelet F., Breaban F., Defer D. (2014). Steel detection in reinforced concrete wall by microwave infrared thermography. NDT E Int..

[97] Khan F., Bolhassani M., Kontsos A., Hamid A., Bartoli I. (2015). Modeling and experimental implementation of infrared thermography on concrete masonry structures. Infrared Phys. Technol..

[98] Lian S., Zheng k., Zhao Y., Bi J., Wang C., Huang Y.S. (2023). Investigation the effect of freeze–thaw cycle on fracture mode classification in concrete based on acoustic emission parameter analysis. Constr. Build. Mater..

[99] Hiasa S., Birgul R., Matsumoto M., Catbas F.N. (2018). Experimental and numerical studies for suitable infrared thermography implementation on concrete bridge decks. Measurement.

[100] Pedram M., Taylor S., Hamill G., Robinson D., OBrien E.J., Uddin N. (2022). Experimental evaluation of heat transition mechanism in concrete with subsurface defects using infrared thermography. Constr. Build. Mater..

[101] Gu J., Unjoh S. (2021). Image processing methodology for detecting delaminations using infrared thermography in CFRP-jacketed concrete members by infrared thermography. Compos. Struct..

[102] Liu F., Liu J., Wang L. (2022). Asphalt pavement fatigue crack severity classification by infrared thermography and deep learning. Autom. Constr..

[103] Cotič P., Kolarič D., Bosiljkov V.B., Bosiljkov V., Jagličić Z. (2015). Determination of the applicability and limits of void and delamination detection in concrete structures using infrared thermography. NDT E Int..

[104] Barreira E., Almeida R.M.S.F., Ferreira J.P.B. (2017). Assessing the humidification process of lightweight concrete specimens through infrared thermography. Energy Procedia.

[105] Gu J.-C., Unjoh S., Naito H. (2020). Detectability of delamination regions using infrared thermography in concrete members strengthened by CFRP jacketing. Compos. Struct..

[106] Ichi E., Dorafshan S. (2022). Effectiveness of infrared thermography for delamination detection in reinforced concrete bridge decks. Autom. Constr..

[107] Woldeamanuel M.M., Kim T., Cho S., Kim H.-K. (2023). Estimation of concrete strength using thermography integrated with deep-learning-based image segmentation: Case studies and economic analysis. Expert Syst. Appl..

[108] Yumnam M., Gupta H., Ghosh D., Jaganathan J. (2021). Inspection of concrete structures externally reinforced with FRP composites using active infrared thermography: A review. Constr. Build. Mater..

[109] Kulkarni N.N., Dabetwar S., Benoit J., Yu T., Sabato A. (2022). Comparative analysis of infrared thermography processing techniques for roadways’ sub-pavement voids detection. NDT E Int..

[110] Mahmoodzadeh M., Gretka V., Mukhopadhyaya P. (2023). Challenges and opportunities in quantitative aerial thermography of building envelopes. J. Build. Eng..

[111] Xie L., Zhu X., Liu Z., Liu X., Wang T., Xing J. (2020). A rebar corrosion sensor embedded in concrete based on surface acoustic wave. Measurement.

[112] Pour-Ghaz M., Kim J., Nadukuru S.S., O’Connor S.M., Michalowski R.L., Bradshaw A.S., Green R.A., Lynch J.P., Poursaee A., Weiss W.J. (2011). Using electrical, magnetic and acoustic sensors to detect damage in segmental concrete pipes subjected to permanent ground displacement. Cem. Concr. Compos..

[113] Guo R., Liu F., Zhang X., Zhao Y., Huang S., Lin X., Yang C. (2023). Feasibility evaluation of the development of type 1-3 acoustic emission sensors for health monitoring of large bridge structures. Ceram. Int..

[114] Liu X., Feng X. (2022). A near-wall acoustic wave-based localization method for broken wires in a large diameter PCCP using an FBG sensor array. Measurement.

[115] Hamdi S.E., Sbartaï Z.-M., Boniface A., Saliba J., Henault J.-M. (2023). Pressure-induced damage monitoring in prestressed concrete of nuclear containment wall segments using acoustic emission technique—Application to VeRCoRs containment building. Eng. Fract. Mech..

[116] Liu F., Guo R., Lin X., Zhang X., Huang S., Yang F., Cheng X. (2022). Monitoring the damage evolution of reinforced concrete during tunnel boring machine hoisting by acoustic emission. Constr. Build. Mater..

[117] Yue J.G., Beskos D.E., Feng C., Wu K. (2022). Hardened fracture characteristics of printed concrete using acoustic emission monitoring technique. Constr. Build. Mater..

[118] Deng H.-S., Fu H.-L., Zhao Y.-B., Shi Y., Huang X. (2022). Using acoustic emission parameters to study damage and fracture characteristics of concrete with different pour intervals cold joints. Theor. Appl. Fract. Mech..

[119] Hou Y., Sun M., Chen J. (2022). Electrical resistance and capacitance responses of smart ultra-high performance concrete with compressive strain by DC and AC measurements. Constr. Build. Mater..

[120] Alrousan R.Z., Alnemrawi B.R. (2022). Punching shear behavior of FRP reinforced concrete slabs under different opening configurations and loading conditions. Case Stud. Constr. Mater..

[121] Fan L., Bao Y., Meng W., Chen G. (2019). In-situ monitoring of corrosion-induced expansion and mass loss of steel bar in steel fiber reinforced concrete using a distributed fiber optic sensor. Compos. Part B Eng..

[122] Kesavan K., Ravisankar K., Parivallal S., Sreeshylam P., Sridhar S. (2010). Experimental studies on fiber optic sensors embedded in concrete. Measurement.

[123] Hong W., Jiang Y., Li B., Qin Z., Hu X. (2018). Nonlinear parameter identification of timber-concrete composite beams using long-gauge fiber optic sensors. Constr. Build. Mater..

[124] Nguyen T.H., Venugopala T., Chen S., Sun T., Grattan K.T.V., Taylor S.E., Basheer P.A.M., Long A.E. (2014). Fluorescence based fibre optic pH sensor for the pH 10–13 range suitable for corrosion monitoring in concrete structures. Sens. Actuators B Chem..

[125] Uva G., Porco F., Fiore A., Porco G. (2014). Structural monitoring using fiber optic sensors of a pre-stressed concrete viaduct during construction phases. Case Stud. Nondestruct. Test. Eval..

[126] Liu Y., Bao Y. (2023). Automatic interpretation of strain distributions measured from distributed fiber optic sensors for crack monitoring. Measurement.

[127] Aulakh D.S., Bhalla S. (2021). 3D torsional experimental strain modal analysis for structural health monitoring using piezoelectric sensors. Measurement.

[128] Wang J., Cao Y., Xiang H., Zhang Z., Liang J., Li X., Ding D., Li T., Tang L. (2022). A piezoelectric smart backing ring for high-performance power generation subject to train induced steel-spring fulcrum forces. Energy Convers. Manag..

[129] Liu X., Yu Y., Li J., Zhu J., Wang Y., Qing X. (2022). Leaky Lamb wave–based resin impregnation monitoring with noninvasive and integrated piezoelectric sensor network. Measurement.

[130] Miao S., Gao L., Tong F., Zhong Y. (2022). Research on high precision optical fiber acoustic emission system for weak damage location on concrete. Constr. Build. Mater..

[131] Demircilioğlu E., Teomete E., Schlangen E., Baeza F.J. (2019). Temperature and moisture effects on electrical resistance and strain sensitivity of smart concrete. Constr. Build. Mater..

[132] Bouzaffour K., Lescop B., Talbot P., Nguyen-Vien G., Gallée F., Rioual S. (2023). Decoupling free chloride and water ingress in concrete by a dielectric resonant sensor. Constr. Build. Mater..

[133] He S., He J., Guo X., Ueda T., Wang Y. (2022). Detection of CFRP-concrete interfacial defects by using electrical measurement. Compos. Struct..

[134] Priou J., Lecieux Y., Chevreuil M., Gaillard V., Lupi C., Leduc D., Rozière E., Guyard R., Schoefs F. (2019). In situ DC electrical resistivity mapping performed in a reinforced concrete wharf using embedded sensors. Constr. Build. Mater..

[135] Jiang B., Wu S. (2023). Resistance measurement for monitoring bending cracks in steel fiber concrete beams test. Alex. Eng. J..

[136] Tafesse M., Alemu A.S., Lee H.K., Cho C.-G., Kim H.-K. (2022). Effect of chloride penetration on electrical resistivity of CNT–CF/cement composites and its application as chloride sensor for reinforced mortar. Cem. Concr. Compos..

[137] Jiao W., Sha A., Liu Z., Jiang W., Hu L., Qin W. (2023). Analytic investigations of snow melting efficiency and temperature field of thermal conductive asphalt concrete combined with electrical-thermal system. J. Clean. Prod..

[138] Zhang J., Heath A., Abdalgadir H.M.T., Ball R.J., Paine K. (2022). Electrical impedance behaviour of carbon fibre reinforced cement-based sensors at different moisture contents. Constr. Build. Mater..

[139] Pei H., Li Z., Zhang B., Ma H. (2014). Multipoint measurement of early age shrinkage in low w/c ratio mortars by using fiber Bragg gratings. Mater. Lett..

[140] Zhang J., Liu C., Sun M., Li Z. (2017). An innovative corrosion evaluation technique for reinforced concrete structures using magnetic sensors. Constr. Build. Mater..

[141] Davis A.M., Mirsayar M.M., Hartl D.J. (2021). A novel structural health monitoring approach in concrete structures using embedded magnetic shape memory alloy components. Constr. Build. Mater..

[142] Tran D.A., Shen X., Sorelli L., Ftima M.B., Brühwiler E. (2023). Predicting the effect of non-uniform fiber distribution on the tensile response of ultra-high-performance fiber reinforced concrete by magnetic inductance-based finite element analysis. Cem. Concr. Compos..

[143] Alabi D.J., Voss M., Ferraro C.C., Riding K., Harley J.B. (2023). Electromagnetic method field test for characterizing steel fibers in ultra-high performance concrete (UHPC). Constr. Build. Mater..

[144] Li Z., Jin Z., Zhao T., Wang P., Li Z., Xiong C., Zhang K. (2019). Use of a novel electro-magnetic apparatus to monitor corrosion of reinforced bar in concrete. Sens. Actuators A Phys..

[145] Xie Z., Zhang D., Ueda T., Jin W. (2022). Fatigue damage analysis of prefabricated concrete composite beams based on metal magnetic memory technique. J. Magn. Magn. Mater..

[146] Yang Z., Li Y., Sang X., Ding Y., Ma B., Chen Q., Kong Q. (2023). Concrete implantable bar enabled smart sensing technology for structural health monitoring. Cem. Concr. Compos..

[147] Loubet G., Sidibe A., Takacs A., Dragomirescu D. (2022). Autonomous Wireless Sensors Network for the Implementation of a Cyber-Physical System Monitoring Reinforced Concrete Civil Engineering Structures. IFAC-PapersOnLine.

[148] Chen J., Li P., Song G., Ren Z. (2016). Piezo-based wireless sensor network for early-age concrete strength monitoring. Optik.

[149] Barroca N., Borges L.M., Velez F.J., Monteiro F., Górski M., Castro-Gomes J. (2013). Wireless sensor networks for temperature and humidity monitoring within concrete structures. Constr. Build. Mater..

[150] Liu G. (2023). A Q-Learning-based distributed routing protocol for frequency-switchable magnetic induction-based wireless underground sensor networks. Future Gener. Comput. Syst..

[151] Siha A., Zhou C. (2023). Experimental study and numerical analysis of composite strengthened timber columns under lateral cyclic loading. J. Build. Eng..

[152] Soltanzadeh F., Edalat-Behbahani A., Pereira E.N.B. (2023). Bond behavior of recycled tyre steel fiber reinforced concrete and basalt fiber-reinforced polymer bars under static and fatigue loading conditions. J. Build. Eng..

[153] Liu C., Wei Y. (2022). Experimental study on interface performance between implantable cement-based sensor and matrix concrete. Constr. Build. Mater..

[154] Gao D., Chen X., Chen G., Zhang L., Zhan Z. (2023). Shear-bond behaviour between concrete and hybrid fibre-reinforced cementitious composites for repairing: Experimental and modelling. J. Build. Eng..

[155] Cheng S., He H., Chen Y., Lan B. (2023). Capacity prediction and crack width calculation of RC beam strengthened with textile and modified concrete. J. Build. Eng..

[156] Ma J., Bai G., Ma H., Bai X., Ni T. (2022). Beam-type experimental study on interfacial bond-slip behavior of steel reinforcement recycled concrete. Constr. Build. Mater..

[157] Li P., Zeng J., Li W., Zhao Y. (2022). Effect of concrete heterogeneity on interfacial bond behavior of externally bonded FRP-to-concrete joints. Constr. Build. Mater..

[158] Pang Y., Wu G., Wang H., Liu Y. (2019). Interfacial bond-slip degradation relationship between CFRP plate and steel plate under freeze-thaw cycles. Constr. Build. Mater..

[159] Bai G., Ma J., Liu B., Chen X. (2020). Study on the interfacial bond slip constitutive relation of I-section steel and fully recycled aggregate concrete. Constr. Build. Mater..

[160] Liu W., Liu C., Liu M., Xu F., Li Z. (2021). Investigation on interfacial properties and calculation models of bamboo scrimber-to-concrete bonding joint. Constr. Build. Mater..

[161] Ding Y., Liu J.-P., Yao G., Wei W., Mao W.-H. (2023). Cyclic bond behavior and bond stress-slip constitutive model of rebar embedded in hybrid fiber reinforced strain-hardening cementitious composites. Constr. Build. Mater..

[162] Ramanathan S., Benzecry V., Suraneni P., Nanni A. (2021). Condition assessment of concrete and glass fiber reinforced polymer (GFRP) rebar after 18 years of service life. Case Stud. Constr. Mater..

[163] Liu M., Cheng X., Li X., Hu J., Pan Y., Jin Z. (2016). Indoor accelerated corrosion test and marine field test of corrosion-resistant low-alloy steel rebars. Case Stud. Constr. Mater..

[164] Soltani A., Nasserasadi K., Ahmadi J., Tafakori E. (2022). Empirical assessment and refinement of corrosion distribution models in the perimeter of corroded steel rebar subjected to chloride ions attack. Case Stud. Constr. Mater..

[165] Li J., Yang E.-H. (2017). Macroscopic and microstructural properties of engineered cementitious composites incorporating recycled concrete fines. Cem. Concr. Compos..

[166] Han X., Wang P., Cui D., Tawfik T.A., Chen Z., Tian L., Gao Y. (2023). Rebar corrosion detection in concrete based on capacitance principle. Measurement.

[167] Fan L., Tan X., Zhang Q., Meng W., Chen G., Bao Y. (2020). Monitoring corrosion of steel bars in reinforced concrete based on helix strains measured from a distributed fiber optic sensor. Eng. Struct..

[168] Ye C., Butler L.J., Elshafie M.Z.E.B., Middleton C.R. (2020). Evaluating prestress losses in a prestressed concrete girder railway bridge using distributed and discrete fibre optic sensors. Constr. Build. Mater..

[169] Liao W., Zhuang Y., Zeng C., Deng W., Huang J., Ma H. (2020). Fiber optic sensors enabled monitoring of thermal curling of concrete pavement slab: Temperature, strain and inclination. Measurement.

[170] Li Q., Yuan L., Ansari F. (2002). Model for measurement of thermal expansion coefficient of concrete by fiber optic sensor. Int. J. Solids Struct..

[171] Pan H.H., Huang M.-W. (2020). Piezoelectric cement sensor-based electromechanical impedance technique for the strength monitoring of cement mortar. Constr. Build. Mater..

[172] Guo Y., Li W., Dong W., Luo Z., Qu F., Yang F., Wang K. (2022). Self-sensing performance of cement-based sensor with carbon black and polypropylene fibre subjected to different loading conditions. J. Build. Eng..

[173] Luo B., Dong J. (2022). Optimizing piezoresistivity of alkali-activated mortar using carboxylated multi-walled carbon nanotubes/basalt fibers. Mater. Lett..

[174] Pan H.H., Guan J.-C. (2022). Stress and strain behavior monitoring of concrete through electromechanical impedance using piezoelectric cement sensor and PZT sensor. Constr. Build. Mater..

[175] Lee M., Mata-Falcón J., Kaufmann W. (2022). Influence of short glass fibres and spatial features on the mechanical behaviour of weft-knitted textile reinforced concrete elements in bending. Constr. Build. Mater..

[176] Chung W.J., Khattak S.H., Cecinati F., Jeong S.-G., Kershaw T., Allen S., Park C.-S., Coley D., Natarajan S. (2023). Resistive and capacitive technology recipes for peak cooling load reductions in the global south. J. Build. Eng..

[177] Ding Y., Liu G., Hussain A., Pacheco-Torgal F., Zhang Y. (2019). Effect of steel fiber and carbon black on the self-sensing ability of concrete cracks under bending. Constr. Build. Mater..

[178] Ling D.C.H., Razak R.A., Yahya Z., Abdullah M.M.A.B., Chaiprapa J., Phan V.T.-A., Mohamed R., Aziz I.H. (2023). Investigation of influence factors and surface treatment on palm oil boiler ash (POBA) based geopolymer artificial aggregate: Impregnation vs. coating method. J. Build. Eng..

[179] Aziz I.H.A., Abdullah M.M.A.B., Razak R.A., Yahya Z., Salleh M.A.A.M., Chaiprapa J., Rojviriya C., Vizureanu P., Sandu A.V., Tahir M.F. (2023). Microstructure, and Porosity Evolution of Fly Ash Geopolymer after Ten Years of Curing Age. Materials.

[180] Ibrahim W.M.A.W., Abdullah M.M.A.B., Jamil N.H., Mohamad H., Salleh M.A.A.M., Sandu A.V., Vizureanu P., Baltatu M.S., Sukmak P. (2023). Alkaline-Activation Technique to Produce Low-Temperature Sintering Activated-HAp Ceramic. Appl. Sci..

[181] Wang L., Aslani F. (2023). Structural performance of reinforced concrete beams with 3D printed cement-based sensor embedded and self-sensing cementitious composites. Eng. Struct..

[182] Lemaire E., Thuau D., Souêtre M., Zgainski L., Royet A., Atli A. (2021). Revisiting two piezoelectric salts within an eco-design paradigm for sensors and actuators applications. Sens. Actuators A Phys..

[183] Zhang H., Wang L., Li J., Kang F. (2023). Embedded PZT aggregates for monitoring crack growth and predicting surface crack in reinforced concrete beam. Constr. Build. Mater..

[184] Xu B., Chen H., Mo Y.-L., Chen X. (2017). Multi-physical field guided wave simulation for circular concrete-filled steel tubes coupled with piezoelectric patches considering debonding defects. Int. J. Solids Struct..

[185] Xu D., Huang S., Cheng X. (2014). Electromechanical impedance spectra investigation of impedance-based PZT and cement/polymer based piezoelectric composite sensors. Constr. Build. Mater..

[186] Du P., Xu D., Huang S., Cheng X. (2017). Assessment of corrosion of reinforcing steel bars in concrete using embedded piezoelectric transducers based on ultrasonic wave. Constr. Build. Mater..

[187] Yan J., Zhou W., Zhang X., Lin Y. (2019). Interface monitoring of steel-concrete-steel sandwich structures using piezoelectric transducers. Nucl. Eng. Technol..

[188] Monazami M., Sharma A., Gupta R. (2022). Evaluating performance of carbon fiber-reinforced pavement with embedded sensors using destructive and non-destructive testing. Case Stud. Constr. Mater..

[189] Zhai Q., Zhang J., Xiao J., Du G., Huang Y. (2021). Feasibility of piezoceramic transducer-enabled active sensing for the monitoring cross-shaped concrete filled steel tubular (CCFST) columns under cyclic loading. Measurement.

[190] Cao P., Zhang S., Wang Z., Zhou K. (2023). Damage identification using piezoelectric electromechanical Impedance: A brief review from a numerical framework perspective. Structures.

[191] Xie J., Yang H., Jing Z., Zhang Y., Hong W., Hu X. (2022). Condition assessment of concrete piers subjected to impact load using fiber optic sensing. Case Stud. Constr. Mater..

[192] Mahjoubi S., Tan X., Bao Y. (2022). Inverse analysis of strain distributions sensed by distributed fiber optic sensors subject to strain transfer. Mech. Syst. Signal Process..

[193] Yan M., Tan X., Mahjoubi S., Bao Y. (2022). Strain transfer effect on measurements with distributed fiber optic sensors. Autom. Constr..

[194] Jayawickrema U.M.N., Herath H.M.C.M., Hettiarachchi N.K., Sooriyaarachchi H.P., Epaarachchi J.A. (2022). Fibre-optic sensor and deep learning-based structural health monitoring systems for civil structures: A review. Measurement.

[195] Li M., Feng X., Han Y. (2022). Brillouin fiber optic sensors and mobile augmented reality-based digital twins for quantitative safety assessment of underground pipelines. Autom. Constr..

[196] Zhang X., Broere W. (2022). Sensing fiber selection for point displacement measuring with distributed optic fiber sensor. Measurement.

[197] Shaikh A., Butler L.J. (2022). Self-sensing fabric reinforced cementitious matrix systems for combined strengthening and monitoring of concrete structures. Constr. Build. Mater..

[198] Zeng M., Chen H., Ling J., Zhao H., Wu D. (2022). Monitoring of prestressing forces in cross-tensioned concrete pavements during construction and maintenance based on distributed optical fiber sensing. Autom. Constr..

[199] Zdanowicz K., Marx S. (2022). Flexural behaviour of thin textile reinforced concrete slabs enhanced by chemical prestressing. Eng. Struct..

[200] Raju B., Kumar R., Senthilkumar M., Sulaiman R., Kama N., Dhanalakshmi S. (2022). Humidity sensor based on fibre bragg grating for predicting microbial induced corrosion. Sustain. Energy Technol. Assess..

[201] Sakaki T., Lüthi B.F., Vogt T. (2022). Investigation of the emplacement dry density of granulated bentonite mixtures using dielectric, mass-balance and actively heated fiber-optic distributed temperature sensing methods. Geomech. Energy Environ..

[202] Zhu J., Wang C., Yang Y., Wang Y. (2023). Hygro-thermal–mechanical coupling analysis for early shrinkage of cast in situ concrete slabs of composite beams: Theory and experiment. Constr. Build. Mater..

[203] Li D., Nie J.-H., Wang H., Yan J.-B., Hu C.-X., Shen P. (2023). Damage location, quantification and characterization of steel-concrete composite beams using acoustic emission. Eng. Struct..

[204] Wang Y., Li P., Liu H., Wang W., Liu Y., Wang L. (2023). Multiple laboratory characterization methods to identify the D-Load of reinforced concrete pipes based on three edge bearing tests. Constr. Build. Mater..

[205] Tailhan J.-L., Rastiello G., Renaud J.-C., Boulay C. (2023). An experimental test for gas pressure measurement within a realistic crack in concrete. Nucl. Eng. Des..

[206] Martinelli F.R.B., Ribeiro F.R.C., Marvila M.T., Monteiro S.N., da Costa Garcia Filho F., de Azevedo A.R.G. (2023). A Review of the Use of Coconut Fiber in Cement Composites. Polymers.

[207] Boumaaza M., Belaadi A., Bourchak M., Juhany K.A., Jawaid M., Marvila M.T., de Azevedo A.R.G. (2023). Optimization of flexural properties and thermal conductivity of Washingtonia plant biomass waste biochar reinforced bio-mortar. J. Mater. Res. Technol..

[208] Du W., Qian C., Xie Y. (2023). Demonstration application of microbial self-healing concrete in sidewall of underground engineering: A case study. J. Build. Eng..

[209] Ji Y., Chen A., Chen Y., Han X., Li B., Gao Y., Liu C., Xie J. (2023). A state-of-the-art review of concrete strength detection/monitoring methods: With special emphasis on PZT transducers. Constr. Build. Mater..

[210] Jayakumari B.Y., Swaminathan E.N., Partheeban P. (2023). A review on characteristics studies on carbon nanotubes-based cement concrete. Constr. Build. Mater..

[211] Geballa-Koukoula A., Ross G.M.S., Bosman A.J., Zhao Y., Zhou H., Nielen M.W.F., Rafferty K., Elliott C.T., Salentijn G.I.J. (2023). Best practices and current implementation of emerging smartphone-based (bio)sensors—Part 2: Development, validation, and social impact. TrAC Trends Anal. Chem..

[212] Tayeh B.A., Ahmed S.M., Hafez R.D.A. (2023). Sugarcane pulp sand and paper grain sand as partial fine aggregate replacement in environment-friendly concrete bricks. Case Stud. Constr. Mater..

[213] Bayrak B., Mostafa S.A., Öz A., Tayeh B.A., Kaplan G., Aydın A.C. (2023). The effect of clinker aggregate on acid resistance in prepacked geopolymers containing metakaolin and quartz powder in the presence of ground blast furnace slag. J. Build. Eng..

[214] Lin G.L., Lin A.X., Liu M.Y., Ye X.Q., Lu D.W. (2022). Barium titanate–bismuth ferrite/polyvinylidene fluoride nanocomposites as flexible piezoelectric sensors with excellent thermal stability. Sens. Actuators A Phys..

[215] Shilar F.A., Ganachari S.V., Patil V.B. (2022). Advancement of nano-based construction materials-A review. Constr. Build. Mater..

[216] Shilar F.A., Ganachari S.V., Patil V.B., Javed S., Khan T.M.Y., Baig R.U. (2022). Assessment of Destructive and Nondestructive Analysis for GGBS Based Geopolymer Concrete and Its Statistical Analysis. Polymers.

[217] Shilar F.A., Ganachari S.V., Patil V.B. (2022). Investigation of the effect of granite waste powder as a binder for different molarity of geopolymer concrete on fresh and mechanical properties. Mater. Lett..

[218] Zheng Y., Zhang Y., Zhuo J., Zhang P., Hu S. (2023). Mesoscale synergistic effect mechanism of aggregate grading and specimen size on compressive strength of concrete with large aggregate size. Constr. Build. Mater..

[219] Zhang P., Sun X., Wang F., Wang J. (2023). Mechanical Properties and Durability of Geopolymer Recycled Aggregate Concrete: A Review. Polymers.

[220] Shilar F.A., Ganachari S.V., Patil V.B., Khan T.M.Y., Khadar S.D.A. (2022). Molarity activity effect on mechanical and microstructure properties of geopolymer concrete: A review. Case Stud. Constr. Mater..

[221] Shilar F.A., Ganachari S.V., Patil V.B., Khan T.M.Y., Javed S., Baig R.U. (2022). Optimization of Alkaline Activator on the Strength Properties of Geopolymer Concrete. Polymers.

[222] Shilar F.A., Ganachari S.V., Patil V.B., Reddy I.N., Shim J. (2023). Preparation and validation of sustainable metakaolin based geopolymer concrete for structural application. Constr. Build. Mater..

[223] Shilar F.A., Ganachari S.V., Patil V.B., Khan T.M.Y., Almakayeel N.M., Alghamdi S. (2022). Review on the Relationship between Nano Modifications of Geopolymer Concrete and Their Structural Characteristics. Polymers.

[224] Bong S.H., Nematollahi B., Xia M., Ghaffar S.H., Pan J., Dai J.-G. (2022). Properties of additively manufactured geopolymer incorporating mineral wollastonite microfibers. Constr. Build. Mater..

